# Ice-nucleating proteins are activated by low temperatures to control the structure of interfacial water

**DOI:** 10.1038/s41467-021-21349-3

**Published:** 2021-02-19

**Authors:** Steven J. Roeters, Thaddeus W. Golbek, Mikkel Bregnhøj, Taner Drace, Sarah Alamdari, Winfried Roseboom, Gertjan Kramer, Tina Šantl-Temkiv, Kai Finster, Jim Pfaendtner, Sander Woutersen, Thomas Boesen, Tobias Weidner

**Affiliations:** 1 Department of Chemistry, Aarhus University, Aarhus C, Denmark; 2 Van ‘t Hoff Institute for Molecular Sciences, University of Amsterdam, Amsterdam, The Netherlands; 3 Department of Molecular Biology and Genetics, Aarhus University, Aarhus C, Denmark; 4 Department of Chemical Engineering, University of Washington, Seattle, WA USA; 5 Swammerdam Institute for Life Sciences, University of Amsterdam, Amsterdam, The Netherlands; 6 Department of Biology, Aarhus University, Aarhus C, Denmark; 7 The Stellar Astrophysics Centre – SAC, Department of Physics and Astronomy, Aarhus University, Aarhus C, Denmark; 8 Interdisciplinary Nanoscience Center – iNano, Aarhus University, Aarhus C, Denmark

**Keywords:** Biophysical chemistry, Surface chemistry

## Abstract

Ice-nucleation active (INA) bacteria can promote the growth of ice more effectively than any other known material. Using specialized ice-nucleating proteins (INPs), they obtain nutrients from plants by inducing frost damage and, when airborne in the atmosphere, they drive ice nucleation within clouds, which may affect global precipitation patterns. Despite their evident environmental importance, the molecular mechanisms behind INP-induced freezing have remained largely elusive. We investigate the structural basis for the interactions between water and the ice-nucleating protein InaZ from the INA bacterium *Pseudomonas syringae*. Using vibrational sum-frequency generation (SFG) and two-dimensional infrared spectroscopy, we demonstrate that the ice-active repeats of InaZ adopt a β-helical structure in solution and at water surfaces. In this configuration, interaction between INPs and water molecules imposes structural ordering on the adjacent water network. The observed order of water increases as the interface is cooled to temperatures close to the melting point of water. Experimental SFG data combined with molecular-dynamics simulations and spectral calculations show that InaZ reorients at lower temperatures. This reorientation can enhance water interactions, and thereby the effectiveness of ice nucleation.

## Abstract (web summary)

Ice-nucleating proteins promote ice formation at high sub-zero temperatures, but the mechanism is still unclear. The authors investigate a model ice-nucleating protein at the air-water interface using vibrational sum frequency generation spectroscopy and simulations, revealing its reorientation at low temperatures, which increases contact with water molecules and promotes their ordering.

## Introduction

Ice-nucleation active (INA) bacteria are the most effective ice nucleators known^1–3^. Using ice-nucleating proteins (INPs) attached to their outer membrane, they promote ice growth at high sub-zero temperatures to gain access to nutrients from plants^4^. INA bacteria can cause severe frost damage to crops and other types of vegetation. Water and ice are fundamental for life and INA bacteria play important roles in this context as they affect interfacial water, the hydrological cycle, local and global climate, and vegetation^5–7^. Recent field studies have revealed large amounts of INA bacteria at high altitudes^8^, which have been identified as an important cause of cloud glaciation, snow and hail precipitation, and cloud formation^5,6,9^. Pure water droplets in the atmosphere can remain supercooled and liquid down to ~−40 °C. It has been shown that, in the presence of the ice-active bacterium *Pseudomonas syringae*, water freezes at temperatures as high as −2 °C^1,10^. INA bacteria may therefore play an important role especially in mixed-phase, low-altitude clouds, where abiotic particles are far less effective ice-nucleating particles than bacteria^1,11^. With their record-holding ice activity, *P. syringae* has also caught attention for technological applications such as artificial snow formation, food preservation, cryo-medicine, and freezing technologies^2,12–14^.

**Fig. 1:**
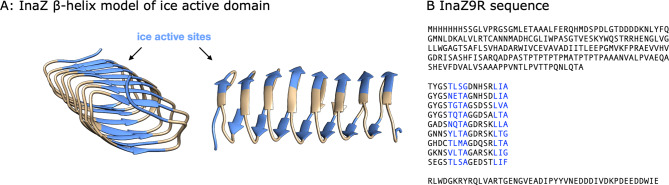
The InaZ repeat sequence and the model InaZ9R construct. Based on current models, the blue sequences are ice-nucleation active sites. **A** Repeat structure of the ice-nucleation active protein InaZ without the N- and C-terminal domains. **B** Sequence of the full InaZ9R construct. It contains an N-terminal tag, the membrane-binding N-terminal domain, the ice-active repeat units, and a C-terminal domain of unknown function^15,17,61,62^.

Despite the numerous environmental and economic aspects where bacterial ice nucleation is of critical importance^4,7^, the fundamental mechanisms of protein-driven ice nucleation are still not clear. The most studied model system is InaZ, the INP of *P. syringae*. Although the InaZ sequence (containing ~1200 residues) has been known for over 30 years^15,16^, and there have been several theoretical studies of INP folding^17–20^, only limited experimental information on the structure of INPs is available^16,21–26^. The current picture of the structure of INPs and their interaction with water is still based on theoretical models and molecular-dynamics (MD) simulations^17,27,28^, with contradicting conclusions. The first model was put forward by Kajava and Lindow and is based on parallel β-sheets^19^. Later, based on homology modeling, Graether and coworkers proposed that InaZ folds into a β-helical structure (Fig. 1), similar to insect antifreeze proteins^17,27,29^.

Ice nucleation takes place at the very interface between single layers of protein interacting with only a few layers of water molecules. The current lack of experimental data about INP activity can be traced back to the general challenges involved in studying the conformation of proteins and water at such thin interfaces. Vibrational sum-frequency generation (SFG) spectroscopy allows one to probe the structure of InaZ at the air–water interface as a model system for the cell–water interface. SFG relies on the resonant enhancement of frequency mixing between visible and infrared laser pulses when the infrared pulse is resonant with a surface vibration^30^. The selection rules of SFG dictate that only ordered species at an interface are detected. Therefore, in the amide-I region, SFG can probe the structure of interfacial proteins and in the water stretching region one can use the technique to observe water molecules interacting with the protein interface.

Previous SFG studies have shown that *P. syringae* and INPs anchored to the surface of hollow *Escherichia coli* cell envelopes can order water at the bacterial surface^31–33^, and that water interaction is enhanced close to the water melting point^31,34^. However, the molecular basis for water ordering and the mechanism behind the activation at low temperatures remain unclear. Bacterial cell surfaces contain a variety of different proteins and other biomolecules and therefore these experiments, performed with entire *P. syringae* cells, could not identify the molecular origin of water ordering.

Here, we report on experiments with isolated model INPs. We use a truncated version of InaZ that contains 9 out of 67 repeat units (repeats 1–4 and 63–67) of the native INA protein (see Fig. 1) of *P. syringae* embedded in the native N- and C-terminal domains. Although the truncation may lead to a reduced ice-nucleation activity, the strategy behind the design has been to provide a native environment for the INA repeat units. For this construct (termed InaZ9R), we show that the central repetitive region of InaZ adopts a β-helical structure and provide conclusive evidence for the ordering of water molecules by INPs. In addition, our data indicate that InaZ reorients at low temperatures and thereby increases contact to water molecules, which increases ice-nucleation activity.

## Results and discussion

### Water structure at the InaZ interface

We investigate the interaction of water in direct contact with InaZ9R with SFG spectroscopy in the water region. Heavy water was used in the experiments for two reasons. First, the vibrational spectrum of D_2_O is narrower than that of H_2_O and can therefore be probed more conveniently using broadband SFG. Second, using D_2_O avoids any overlap of water-bending modes with the amide-I region.

**Fig. 2:**
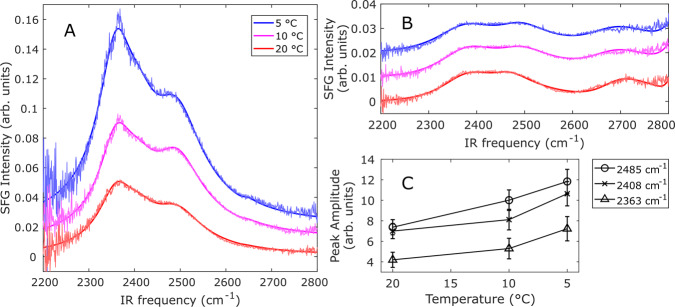
Temperature-dependent OD-stretch SFG spectra collected in the ssp polarization combination, along with fits. For clarity, the 10 °C and 5 °C spectra are offset from the corresponding 20 °C spectrum by 0.01 and 0.02 arbitrary units, respectively. **A** Spectra of water molecules interacting with the ice-nucleating active protein InaZ9R in D_2_O-based PBS buffer. The intensity of the water signal is increased significantly for lower temperatures in the presence of inaZ9R. **B** Spectra of the neat buffer surfaces remain essentially unchanged. **C** Plot of the SFG amplitudes for the different water modes fitted to the InaZ9R spectra.

Figure 2 displays temperature-dependent SFG spectra collected with the ssp (s-polarized SFG, s-polarized visible, and p-polarized infrared) polarization combination for InaZ9R in phosphate-buffered saline (PBS) buffer at the air–water interface (panel A) and for neat PBS buffer (panel B). For both the InaZ and pure buffer spectra, resonances corresponding to O–D stretching and free O–D are observed; however, the presence of the InaZ at the interface results in an extra resonance near 2408 cm^−1^, probably related to water molecules interacting with InaZ9R^35^. N–H resonances originating from protein amine groups can in principle also contribute to the spectra. However, as hydrophilic side chains tend to remain disordered at hydrophobic surfaces such as the air–water interface^36^, at most minor contributions are expected. For PBS, there is also a weak, but discernible signature of the free O–D resonance near 2655 cm^−1^. Already at 20 °C, the intensities of the observed resonances are significantly stronger when InaZ9R is present, compared to the neat buffer spectrum, indicating an increased water order. This is often observed for biological interfaces, such as protein films and lipid monolayers, and can be explained by the interaction and alignment of water molecules at the InaZ9R interface^37,38^. As the buffer temperature is decreased to 10 °C, and subsequently to 5 °C, the water signal is strongly increased. Figure 2C summarizes the intensity changes of the different water modes (see Table S3 for fitting parameters). Potential N–D contributions from the backbone, the so-called amide-A modes, or from side chains may also contribute to this spectral region as a narrow peak at ~2550 cm^−1^ ^39^. Although the N–D-modes may potentially affect a ~100 cm^−1^ region, the whole O–D region (~500 cm^−1^ broad) exhibits an increasing SFG signal for decreasing temperatures, thus making it probable that the influence of the N-D modes on the spectra is rather limited. Temperature changes can potentially affect the density of interfacial protein layers and thereby influence the water signal. We therefore determined the protein coverage of the air–water interface at 5, 10, and 20 °C with X-ray photoelectron spectroscopy. This data, recorded for InaZ9R lifted off the air–water interface, showed that the surface coverage was largely unaffected by the temperature change (see Supplementary Note 1).

SFG spectra of the neat PBS buffer surface do not change appreciably with temperature. This is in agreement with previous studies of non-ice-nucleating interfaces^31,40^. Temperature-dependent SFG experiments with model lipid monolayers, non-INPs such as lysozyme and lysed ice bacteria have been used to test whether the increased water order is indeed related to ice activity. In those reports, an increase of the water signal has only been observed for ice-active proteins, which showed that the increased water order at lower temperatures is related to INPs and not a generic property of aqueous biological interfaces^31^. For the INP InaZ9R, we clearly observe effective water ordering that becomes more efficient at temperatures near its “operating temperature”—close to the water melting point at which INPs are typically active. The similarity of this behavior to what has been observed for ice bacteria provides strong experimental evidence that InaZ is indeed the water-ordering agent at the surface of *P. syringae* cells.

### InaZ secondary structure in solution

This raises the question as to what is the structural basis for the enhanced water order at lower temperatures. Previous studies with INA bacterial cells have not been able to answer this question because of the structural heterogeneity of bacterial cell surfaces. With the InaZ9R construct, we now attempt to address this question—by firstly determining the solution-state structure of InaZ9R and thereafter resolving the binding geometry of InaZ9R at the air–water interface.

**Fig. 3:**
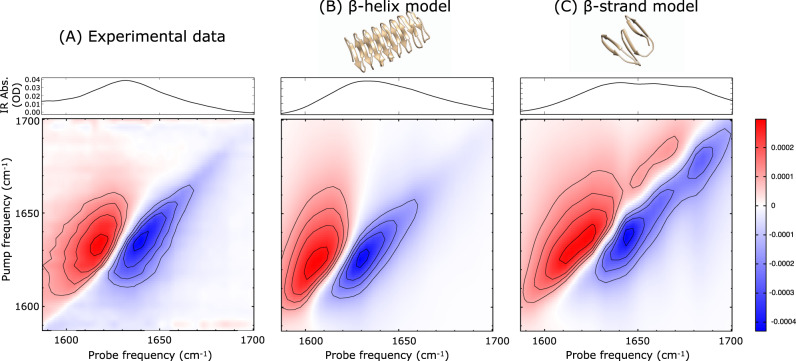
Comparison between experimental and calculated FT-IR and 2D-IR spectra of InaZ in solution. **A** Experimental, and (**B**, **C**) calculated spectra, with the FT-IR spectra shown on top and the 2D-IR spectra below. The IR spectra are sensitive to the secondary structure of proteins^41^ and can be regarded as a fingerprint of their conformation. The comparison between the experimental and calculated spectra shows that the β-helix model captures the experimental data very well, whereas the β-sheet model shows significant deviations. The 2D-IR spectra are recorded with a pump polarization that is parallel with respect to the probe polarization. Color bar indicates differential absorption (in ΔOD) for all 2D-IR spectra.

To determine the secondary structure in solution, we use Fourier-transform infrared (FT-IR) and two-dimensional infrared (2D-IR) spectroscopy. Figure 3A displays the experimental FT-IR and 2D-IR amide-I (1600–1700 cm^−1^) spectra of InaZ9R in PBS buffer. Two-dimensional IR spectra can be recorded by exciting the sample with a narrowband pump pulse with a given frequency ω_pump_, after which the IR absorbance is recorded across the whole spectral range with a broadband probe pulse. By plotting the differential absorbance with versus without the pump excitation as a function of ω_probe_ and the scanned ω_pump_, 2D-IR spectra can be obtained, which can be regarded as the vibrational analog of 2D-NMR spectra^41^. If two modes, A and B, are coupled, the absorption of one is affected when the other is excited. So, if mode A is excited by the pump beam (ω_pump_ = ω_A_), then an absorption change will be recorded with the probe beam at ω_probe_ = ω_B_, and vice versa, which gives rise to a distinct cross-peak pattern at (ω_A_, ω_B_) and (ω_B_, ω_A_) in the 2D-IR spectrum. In the case of the amide-I mode of proteins, the individual cross-peaks can generally not be resolved, but the shape of the amide-I 2D-IR spectrum is still very sensitive to the secondary structure^42^. The FT-IR and 2D-IR spectra of InaZ9R are composed of a single broadband that peaks at 1632 cm^−1^, and the 2D-IR spectrum does not contain any strong cross-peaks. The spectra show a close resemblance to previously measured IR spectra of proteins composed mainly of β-helices, with segments that contain other secondary structures^43^. To test which of the theoretical models agrees best with the experimental data, we calculate 2D-IR spectra based on both the β-sheet and the β-helix model to compare with the experimental data (Fig. 3). For the β-sheet model, we base the calculation on the published 48-mer repeat unit composed of stacked antiparallel β-sheets^19^. For the β-helix model, we use a structural model that was proposed in a previous INP simulation study^17^.

The models are briefly equilibrated using a 100 ns molecular-dynamics (MD) simulation in GROMACS. The 2D-IR spectra are calculated with an amide-I Hamiltonian model (see Supplementary Notes 2 and 6 for details of the simulations and spectral calculations). To calculate the spectra depicted in Fig. 3B and C, an additional inhomogeneous broadening was applied to the local-mode frequencies in order to match the broad experimental peak shape. This is probably due to the C- and N-termini of the full InaZ9R sequence used in the experiments (Fig. 1) that are likely to adopt a less-well defined structure in solution, as is also indicated by UV-circular dichroism (CD) spectroscopy (Supplementary Figure 5 and Note 3). It is clear that the β-helical model results in a closer spectral match than the β-sheet model. The calculated β-sheet model spectra are significantly shifted with respect to the experimental resonance positions, and exhibit high-frequency peaks that are absent in the experimental data. The 2D-IR spectra calculated for the β-helix model, on the other hand, capture the experimental spectra very well. Minor deviations of experiment and calculation observed for the β-helix model might be explained by spectral contributions of coiled structures in the N- and C-terminal domains that are different from the purely random-coil structure (here modeled by the included inhomogeneous broadening). In summary, we conclude that the β-helix model agrees with the IR data and describes the solution structure of InaZ9R well.

IR spectra recorded at different temperatures show that the secondary structure of InaZ9R does not change as a function of temperature (see Supplementary Figure 8 and Note 4), which indicates that the observed temperature-dependent water ordering (Fig. 2) is caused by another effect.

### InaZ reorientation at low temperatures

**Fig. 4:**
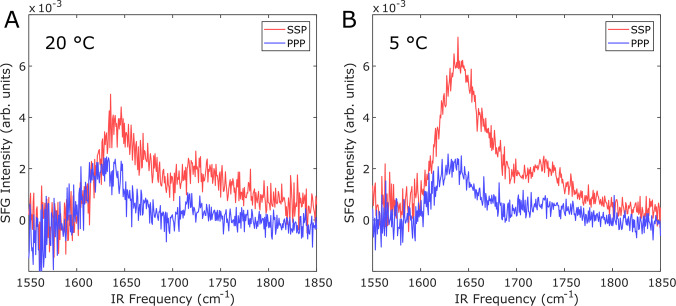
Temperature-dependent amide-I SFG spectra of InaZ9R at the air–water interface in ssp and ppp polarization. **A** Spectra recorded at 20 °C show a resonance near 1630 cm^−1^ related to β-sheet type structure and a resonance near 1720 cm^−1^ related to lipids. **B** Spectra collected for 5 °C show a strong increase in amide-I ssp intensity, which indicates the protein has changed orientation at the interface.

To determine the structure of InaZ9R at the water interface and how the protein can promote water ordering at low temperatures, we record amide-I SFG spectra of InaZ9R at the air–water interface. Amide-I SFG spectra are sensitive to secondary structure, and can also provide information about the orientation of interfacial proteins^30^. Fig. 4 displays amide-I SFG spectra collected at 20 °C and 5 °C. The spectra recorded at 20 °C show modes centered near 1630 cm^−1^ and 1720 cm^−1^. The 1720 cm^−1^ mode can be assigned to lipid molecules, which could indicate the presence of protein-associated lipids. Both sodium dodecyl-sulfate polyacrylamide gel electrophoresis and intact-protein mass spectrometry point to an increased molecular weight of the protein compared with the expected mass based on its assumed amino-acid sequence (see Supplementary Note 3). It is also possible that the mode is due to protonated aspartic acid and glutamic acid side chain modes. While these sites are typically deprotonated at neutral pH, the pH and protonation state within the interior of proteins/protein layers can differ significantly from the pH of the surrounding solution^44–46^. Based on literature about infrared absorption resonances and SFG studies, the feature at ~1630 cm^−1^ can be assigned to β-sheet type protein structures^47–49^ (see Supplementary Note 5 for details about the spectral fits). Since only ordered structures are visible in SFG spectra, the presence of the amide-I mode shows that InaZ9R forms a well-aligned layer at the air–water interface. This is also supported by SFG spectra in the C–H stretching region, where aliphatic and aromatic modes are visible, which demonstrates a high degree of alignment within the hydrophobic side chains (see Supplementary Figure 8/Supplementary Note 5).

At 5 °C, the resonance positions remain largely unchanged while the peak ratio of the 1630 cm^−1^ protein resonance between the ssp and ppp spectra is dramatically increased. The fact that the resonance positions are similar at both temperatures indicates that the folding of InaZ9R does not change considerably.

**Fig. 5:**
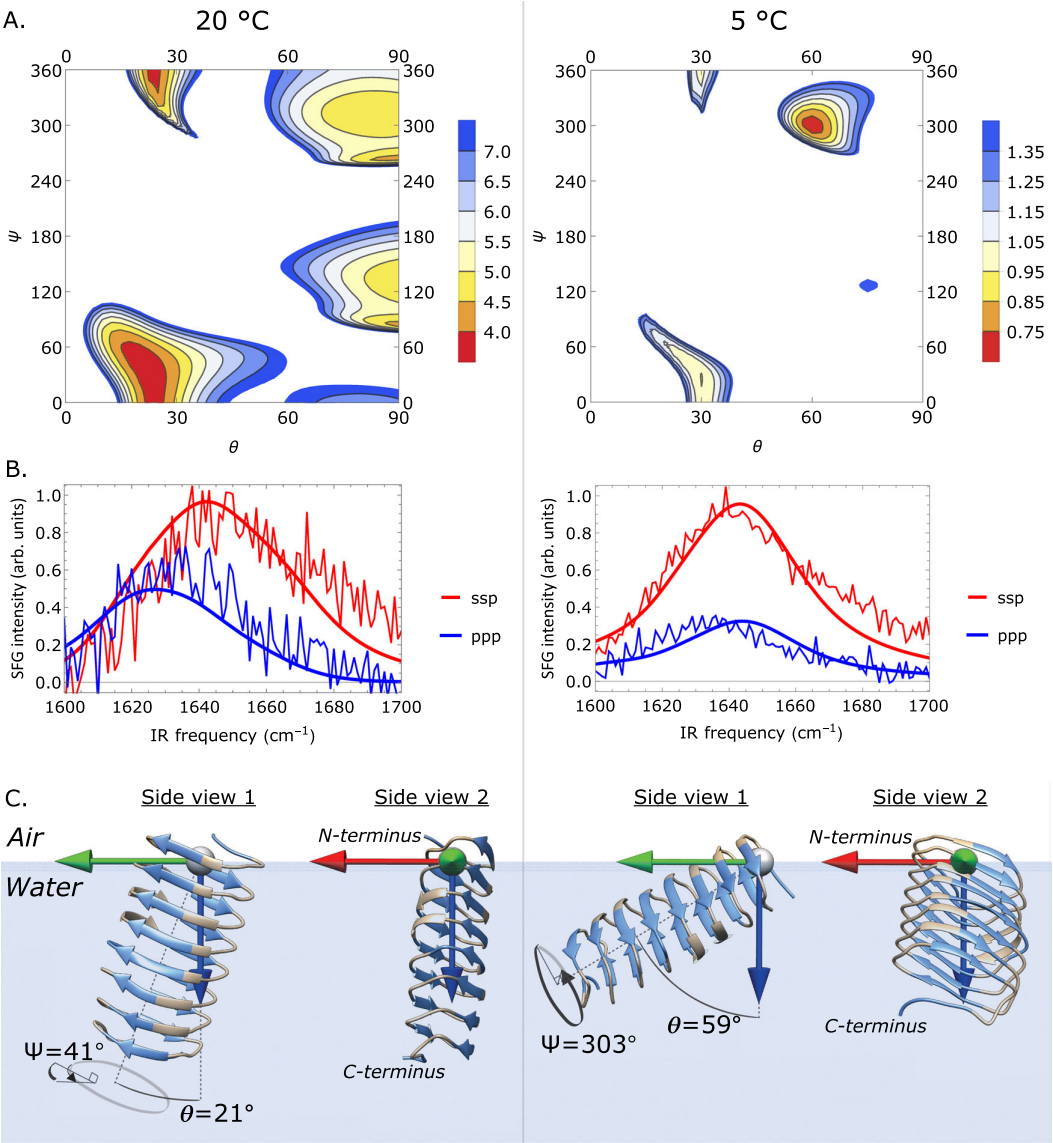
Theoretical and experimental SFG amide-I spectra of InaZ9R at the air–water interface. **A** RSS plots illustrating the match between theory and experiment for different tilt (*θ*) and twist (*Ψ*) angles. **B** Calculated spectra resulting from (*θ*, Ψ) fits starting at the minimum RSS position, resulting in best matching tilt and twist angles of *θ* = 21° ^+31°^_−40°_ and Ψ = 41° ^+72°^_−110°_ for 20 °C, and *θ* = 59° ^+18°^ _−8°_ and Ψ = 303° ^+42°^_−36°_ for 5 °C (positive and negative uncertainties are given as super- and subscripts, see SI). **C** Schematic representation of the best matching protein orientations. InaZ9R reorients at lower temperatures, with a more inclined orientation at 5 °C. In a tightly packed protein layer, the low-temperature pose increases the interaction of the INA sites (marked blue) with water.

Often, a change in amide-I peak ratios indicates a reorientation within protein monolayers. We have calculated SFG spectra based on the IR-derived β-helix model, which encompasses the central repeat domain of InaZ9R^17^. The experimental SFG spectra can be reproduced well by the calculated spectral response of the central repeat domain (see Fig. 5). Based on the selection rule of SFG that only ordered species will generate a signal, this might indicate that the N- and C-terminal domains adopt a coiled or less defined structure at the interface and that their contribution to the measured signal is small. Figure 5a presents an overview of the comparison between calculated and experimental SFG spectra for a range of combinations of tilt and twist angles (*θ*, *Ψ*) with a step size of 2.5°, indicated by the residual sum-of-squares (RSS). There is a narrow range of angles for which theory and experiment closely match. The closest spectral match is found for small tilt angles for 20 °C, whereas the minimal RSS values for 5 °C are found at larger tilt angles. Going from 20 °C to 5 °C, fits that start at the minimal RSS values result in a transition from (*θ*, *Ψ*) = (21°, 45°) to (59°, 290°) (see Fig. 4B). The calculated spectra for these orientations capture both the resonance positions and the relative intensities of the experimental data well. Deviations could be explained by weak contributions by the terminal domains.

In summary, the data indicate a reorientation to a larger tilt angle *θ* and a *Ψ* rotation around the helical axis with decreasing temperature, which changes the orientation of the INA sites considerably (see Fig. 5C). A normal-mode analysis of the two strongest types of modes in the spectrum, the one around 1625 cm^−1^ and the one at ~1670 cm^−1^, reveals that these can be assigned to the B_2_ mode and to β-turns (see Supplementary Figure 3 and Supplementary Note 2), in line with previous literature^49^.

### InaZ structure and activation

The IR data provides experimental evidence that the central repeat region of InaZ has a β-helical fold. In addition, the SFG data support that this conformation is also stable in protein films interacting with interfacial water. Simulations have predicted a β-helical folding motif based on sequence homology with insect AFPs^17,29^. This study now provides evidence that indeed bacterial INPs and insect AFPs have a similar β-helical structure, despite having contrary effects on water molecules. The difference in ice activity is probably caused by the difference in size of the ice-interacting surface—insect AFPs have sizes around 10 kilo-Dalton, whereas INPs have sizes of several mega-Dalton.

**Fig. 6:**
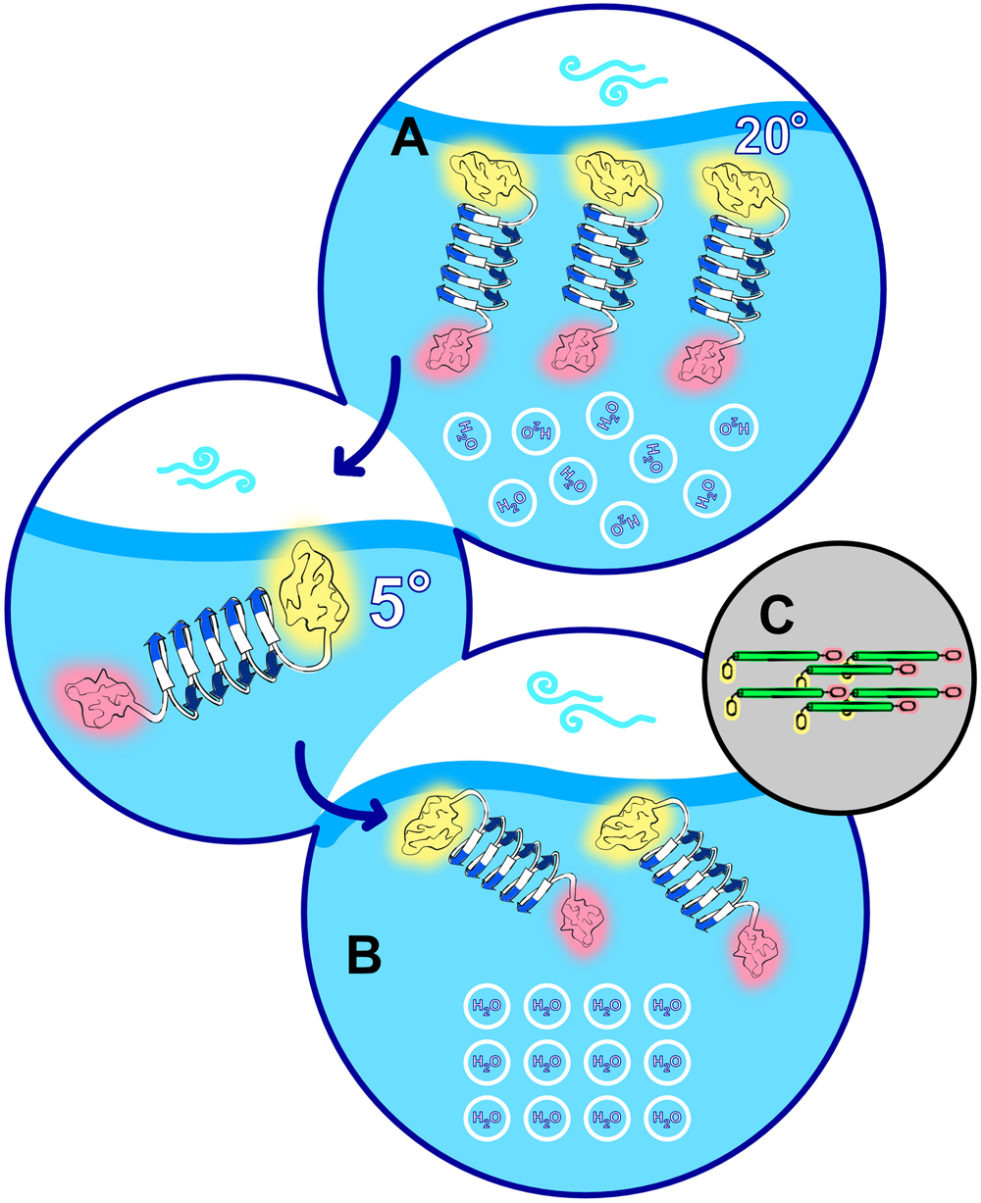
Illustration of the interaction of InaZ9R with water molecules. **A** At room temperature, the ice-nucleating sites (blue arrows, N- and C- terminal domains marked yellow and pink, respectively) are buried in the protein film resulting in relatively low water order. **B** At 5 °C, InaZ9R reorients with the long axis of the helix more parallel to the surface. In this orientation the ice-nucleating sites are exposed to the interfacial water layer. Although the specific water orientation cannot be inferred from homodyned SFG data, the results clearly show increased water order, which promotes ice nucleation. **C** The low-temperature protein pose, with the β-helix (green) axis of InaZ parallel to the surface, and the N- and C termini again marked yellow and pink, supports recent models for INP assembly at bacterial surfaces.

Earlier studies have reported that the ice activity of INA bacteria is increased when the bacteria are exposed to temperatures ~4 °C^50^. The SFG analysis of the interfacial protein structure shows that this effect can be explained by INP reorientation when cooled to lower temperatures. Figure 5c provides an overview of the temperature-induced transition of InaZ9R. Although at room temperature the long axis of the protein is oriented perpendicular to the water surface, at lower temperatures the protein reorients and adopts an orientation that is aligned more parallel with the interface. In addition, InaZ9R rotates along the long axis of the helix, such that the ice-active strands are oriented more parallel to the water surface. As summarized in Fig. 6, the low-temperature geometry increases the exposure of INA sites to the interfacial water network when the protein is part of a densely packed layer. Lateral assembly of InaZ in the room temperature state (Fig. 6A) will lead to burial of INA sites within the protein film, while at low temperatures (Fig. 6B) the protein film exposes a large area of INA sites to the water. Evidently, the reorientation of InaZ at lower temperatures enhances water ordering and is thereby the probable cause of the observed increase of SFG water signal at low temperatures^51^. At cell surfaces, based on current models, InaZ is anchored through its N-terminal domain^17^ and would presumably be flexible enough to reorient from perpendicular to parallel to the cell surface with decreasing temperatures. Similar to the air–water interface studied here, InaZ assembles into large patches at the cell surface. Such a surface assembly maximizes the size of the ice-nucleation site and thereby increases the ice-nucleation activity. The current picture of the assembly of InaZ at the bacterial surface with the long protein axis parallel to the surface (Fig. 6C) is in excellent agreement with the low-temperature pose we identified with SFG, where the long helix axis is oriented parallel to the air–water interface. InaZ9R is a model INP with a significantly shortened ice-active domain. Therefore, the extent of water interactions may differ from the native INP. Yet, based on the present data, we can conclude that the reason why water close to ice-active bacterial cells becomes more ordered when the water is cooled to lower temperatures is likely not to be found in changes of INA sites as such, but is driven by a reorientation of InaZ into a “flat”, carpet-like geometry at a lower temperature, which maximizes the availability of ice-nucleation sites to water.

The reorientation of InaZ9R could be caused by a subtle shift in the sensitive balance of forces between protein side chains, water molecules and lateral protein interactions. In principle such reorientations are independent of ice activity and could occur also in other protein films. For ice-active proteins these effects could be more pronounced because studies of INPs and related AFP β-helices have shown that for this class of proteins all of these factors are affected by temperature. For example, it has been shown that their hydration^52^ and lateral protein interactions^53^ change significantly when decreasing the temperature close to the water melting point. The protein–water interface  of INPs has also been observed to become less dynamic at lower temperatures^28^. Together, these factors can alter protein–protein as well as protein–water contacts and impact the lateral organization of InaZ at interfaces.

In summary, the presented data provides direct experimental evidence that InaZ adopts a β-helical fold in solution and in protein layers in contact with water. Furthermore, the data supports that InaZ is indeed the driving force for the water ordering at lower temperatures observed for *P. syringae*. InaZ reorients at the interface when cooled from room temperature to the “operating temperature” of INPs, leading to an increased exposure of the INA sites. This temperature-induced activation leads to an increased water order and, consequently, surface freezing.

## Methods

### Protein purification

The truncated InaZ protein construct (termed InaZ9R), containing nine repeat units, was prepared following protocols developed for a related 16 repeat InaZ construct^54^. The InaZ9R sequence was cloned into the pET30 Ek/LIC vector (Novagen, Merck Biosciences) according to manufacturer’s instructions, and used for transformation of *E. coli* Rosetta (DE3) competent cells. A large-scale culture was grown in lysogeny broth (LB) medium and induced with 1 mM isopropyl β-d-1-thiogalactopyranoside overnight at 20 °C. The protein was purified by immobilized metal affinity chromatography. Fractions containing InaZ9R were subsequently loaded on an anion exchange chromatography column attached to a fast protein liquid chromatography (FPLC) system and finally on a size exclusion chromatography column attached to an FPLC system. The individual fractions were either flash-frozen in liquid nitrogen and stored for later use in the size exclusion buffer (20 mM Tris-HCl pH 7.5; 150 mM NaCl), or buffer-exchanged into (D_2_O) PBS buffer for further characterization. Further details on materials and primers used are provided in Supplementary Note 3. Biological materials are available from the authors upon reasonable request.

### Vibrational spectroscopy

#### Sample preparation

The sample solutions for SFG and IR spectroscopy were prepared using D_2_O-based buffers. Using D_2_O is an established strategy in vibrational protein spectroscopy to prevent overlap of the amide-I modes of the protein backbone with the OH-bending mode of water. In addition, using D_2_O is advantageous in broadband SFG spectroscopy because the water spectrum is narrower, which facilitates the acquisition of spectra representing the entire water region in one experiment. D_2_O forms slightly stronger hydrogen bonds and H_2_O/D_2_O replacement may cause small changes, so the temperature dependencies observed in our experiments might be slightly shifted in D_2_O as compared with H_2_O^55^. At the same time, replacing H_2_O by D_2_O does not result in any qualitative differences, and our conclusions will also apply to the case of H_2_O.

#### 2D-IR

Transmission 2D-IR spectra were recorded by overlapping a chopped narrowband pump IR laser pulse and a broadband probe IR laser pulse in the sample with a delay of 1.5 ps^56^. An additional reference beam, identical to the probe, was passed through the sample at a few mm displacement. The difference-absorption spectrum between the unchopped and chopped pulses was recorded on a spectrograph with an IR-sensitive MCT (HgCdTe) array, and corrected for peak-to-peak stability with the reference beam. The 7 μL IR samples (prepared at 0.8 mg/mL, 18 μM, in D_2_O PBS buffer at pD = 7.4) were pipetted in between two CaF_2_ windows that were spaced 50 μm apart and sealed by a greased Teflon spacer.

#### SFG

SFG spectra were recorded in standard reflection geometry, by overlapping a tunable broadband IR laser pulse with a narrowband visible (~800 nm) laser pulse in space and time. The generated SFG light was routed to a spectrograph and recorded with an EMCCD camera. All spectra were background subtracted and subsequently normalized by a spectrum from a neat gold surface. Protein samples were prepared at 0.44 mg/mL, 10 μM, in phosphate-buffered D_2_O at pD = 7.4, to avoid spectral interference from water bending modes.

### Spectral calculations

#### 2D-IR

A two-exciton amide-I Hamiltonian was constructed based on the solution-state MD trajectory at room temperature. The couplings in the Hamiltonian were based on the transition-dipole coupling model^57^ for non-nearest neighbors, whose interaction is dominated by through-space effects, while the nearest neighbor couplings, dominated by through-bond effects, are modeled with a parameterized map of an ab initio calculation with the 6-31 G + (d) basis set and B3LYP-functional^58^. The local-mode frequencies on the diagonal of the Hamiltonian are estimated using the same hydrogen bond-shift model as published previously^59^, which is based on the effect that various types of hydrogen bonds to the amide group have on the amide-I frequency^60^. By diagonalizing the Hamiltonian, we obtain the amide-I eigenvectors and eigenvalues from which the 2D-IR spectra are calculated for each of the frames, after which the response is averaged over all frames.

#### SFG

A one-exciton Hamiltonian was constructed based on the 100 ns-relaxed β-helix model, using the same coupling models as for the 2D-IR calculations. To determine the orientation of the protein from the amide-I ssp and ppp lineshapes and intensities for 5 and 20 °C, we calculated 10,000 spectra as a function of θ and Ψ (with a grid size of 2.5°) and determined the deviation between calculation and experiment for each (θ, Ψ) point. Subsequently, Levenberg-Marquardt least-square fits were performed with the best (θ, Ψ) values as the initial guess.

## Supplementary information

Supplementary Information

## Data Availability

All spectroscopic data are available from the authors upon request and on https://figshare.com under 10.6084/m9.figshare.13372793. Further details about experiments and data analysis can be found in the Supplementary Information document (Supplementary Notes 1–6).

## Code availability

Code used to calculate SFG and 2D-IR spectra is available from the authors upon reasonable request.
