# Supplementary material for: Ice-nucleating proteins are activated by low temperatures to control the structure of interfacial water
Source: Nat Commun. 2021 Feb 19;12:1183. doi: 10.1038/s41467-021-21349-3 (PMC7895962; doi:10.1038/s41467-021-21349-3)
Supplement: Supplementary file 1 — Supplementary Information [file 41467_2021_21349_MOESM1_ESM.pdf]

Supplementary Information for:

Ice Nucleating Proteins Are Activated by Low Temperatures to Control the Structure of Interfacial Water

Steven J. Roeters,[1,2,‡] Thaddeus W. Golbek,[1,‡] Mikkel Bregnhøj,[1,‡] Taner Drace,[3] Sarah Alamdari,[4] Winfried Roseboom,[5] Gertjan Kramer,[5] Tina Šantl-Temkiv,[6,7] Kai Finster,[6,7] Jim Pfaendtner,[4] Sander Woutersen,[2] Thomas Boesen,[3,8] Tobias Weidner[1,4,8,*]

[1] Department of Chemistry, Aarhus University, 8000 Aarhus C, Denmark

[2] Van 't Hoff Institute for Molecular Sciences, University of Amsterdam, 1098 XH Amsterdam, The Netherlands

[3] Department of Molecular Biology and Genetics, Aarhus University, 8000 Aarhus C, Denmark

[4] Department of Chemical Engineering, University of Washington, Seattle, WA 98103, United States

[5] Swammerdam Institute for Life Sciences, University of Amsterdam, 1098 XH Amsterdam, The Netherlands

[6] Department of Biology, Aarhus University, 8000 Aarhus C, Denmark

[7] The Stellar Astrophysics Centre – SAC, Department of Physics and Astronomy, Aarhus University, 8000 Aarhus C, Denmark

[8] Interdisciplinary Nanoscience Center – iNano, Aarhus University, 8000 Aarhus C, Denmark

‡ These authors contributed equally

Corresponding Author

* Tobias Weidner: weidner@chem.au.dk

Table of Supplementary Contents

Supplementary Note 1: X-ray Photoelectron Spectroscopy (XPS) 3
- Supplementary Methods 1 3
- Supplementary Results 1....3

Supplementary Note 2: Spectral Calculations 6
- Supplementary Methods 2 6
  - 2D-IR Spectra 6
  - SFG Spectra 6
- Supplementary Results 2....7
  - The Effect of Inhomogeneous Broadening on Calculated 2DIR Spectra 7
  - Normal-mode Analysis 9

Supplementary Note 3: Sample Preparation and Characterization....10
- Supplementary Methods 3 10
  - Biochemistry 10
  - InaZ Lyophilization of the IR and SFG Samples Assessed with UV-CD 11
  - FT-IR and 2D-IR Sample Preparation 12
  - SFG Sample Preparation....12
- Supplementary Results 3....12
  - Mass Spectral Analysis of Recombinant InaZ....12

Supplementary Note 4: 1D-IR (FT-IR) and 2D-IR Spectroscopy....15
- Supplementary Methods 4 15
  - 1D-IR (FT-IR) Spectroscopy 15
  - 2D-IR Spectroscopy....15
- Supplementary Results 4....15
  - Temperature-Dependent IR Spectra 15

Supplementary Note 5: SFG Spectroscopy....16
- Supplementary Methods 5 16
  - SFG Spectroscopy....16
  - SFG Fitting Methods 16
- Supplementary Results 5....16
  - SFG Spectra in the C-H Region....16
  - Peak-Fitting Parameters of the OD-Stretch Spectra 17
  - Peak-Fitting Parameters of the Amide-I Spectra 19

Supplementary Note 6: Molecular-Dynamics Simulations 20
- Supplementary Methods 6 20
- Supplementary Results 6....20

Supplementary References....21

Supplementary Note 1: X-ray Photoelectron Spectroscopy (XPS)

Supplementary Methods 1

XPS experiments were performed on a Kratos AXIS Ultra DLD instrument equipped with a monochromatic Al Kα X-ray source (hν = 1486.6 eV). All spectra were collected in hybrid mode at a take-off angle of 0° (angle between the sample surface plane and the axis of the analyzer lens). The spectra were collected at fresh spots on the sample (n=3) and were charge corrected to the C_{1s} aliphatic carbon binding energy at 285.0 eV, after which a linear background was subtracted for all peak area quantifications. Analyzer pass energy of 160 eV was used for compositional survey scans. High-resolution scans of C_{1s} and N_{1s} elements were collected at an analyzer pass energy of 20 eV. Compositions and fits of the high-resolution scans were produced in CasaXPS.

XPS samples were prepared by Langmuir-Schaeffer (LS) deposition of the INP9R protein from the air/water interface onto silicon substrates. The substrates were cleaned immediately before use by 15 min sonication in dichloromethane (DCM), acetone, and ethanol and dried with nitrogen. The protein was injected at 7 uM in the trough used for SFG experiments and allowed to equilibrate for 30 min at each given temperature before deposition. After deposition, the samples were left to dry overnight in a dark glovebox under a slow stream of nitrogen.

Supplementary Results 1

XPS is a surface analytical technique able to provide precise atomic level compositions of the first approximately 10 nm of a surface.[17, 18, 19] In short, the irradiated sample emits photoelectrons from the core level by transfer of energy from the photon to core-level electrons. The energy of the emitted photoelectron is directly related to the atomic molecular environment of the electron, and the quantity of a specific photoelectron relates to the concentration of a specific element. The depth of XPS analysis is attributed to the inelastic mean free path (IMFP) of a photoelectron emitted; the average distance than a photoelectron travels between successive inelastic collisions. Any photoelectrons that escape the surface with energy loss contribute to the background. All elements, expect for hydrogen and helium, present in quantities greater than 0.1 atomic percent can be identified with XPS. Previously, XPS has been used to determine elemental compositions on flat surfaces as well as monolayer coverage of proteins.[17, 18, 20, 21, 22, 23, 24, 25, 26, 27] In this study, we used XPS to follow the surface coverage of a protein (InaZ9R) at the air-water interface. The protein was removed from the air-water interface by LS depositon with silicon substrates. The samples were then analyzed by XPS for the amount of protein, which directly relates to the amount of protein at the air-water interface. The amount of protein the air-water interface was measured at 5˚C, 10˚C, and 20˚C corresponding to the SFG spectra recorded at these temperatures in the main article. Supplementary Figure 1 shows the survey and high-resolutions spectra of the LS deposition onto substrates and Supplementary Table 1 shows the survey spectrum atomic compositions for all expected elements in each sample. Figure 7B shows the C1s high-resolution spectra, and it is clear from the peak at 288.0 eV that there is protein on the substrate surface. Next, to determine the amount of protein on the surface, we can directly

use the atomic percent N1s in Supplementary Table 1, and since the only source of nitrogen is from the protein, thus it is a direct indication of the relative amount of protein on the substrate surface. Due to the fact that the substrate below the protein is not well measured, likely due to the thickness of the protein and the salt overlayer, the relative amount of protein to salt buffer can be used to indicate the amount of protein on the substrate surface and thus direly related to the air/water surface of our trough experiments. We observe 8.9 ± 0.3, 9.2 ± 0.5, and 9.0 ± 0.7 atomic percent nitrogen for 5˚C, 10˚C, and 20˚C, respectively. Within the experimental error, the nitrogen percent is the same between the temperatures. Furthermore, the counts per second (CPS) measured by XPS in the N1s high-resolution (Supplementary Figure 1C) and also very similar. Together, these two pieces of information from XPS show that the amount on INP9R protein is constant for all temperatures measured in this study.

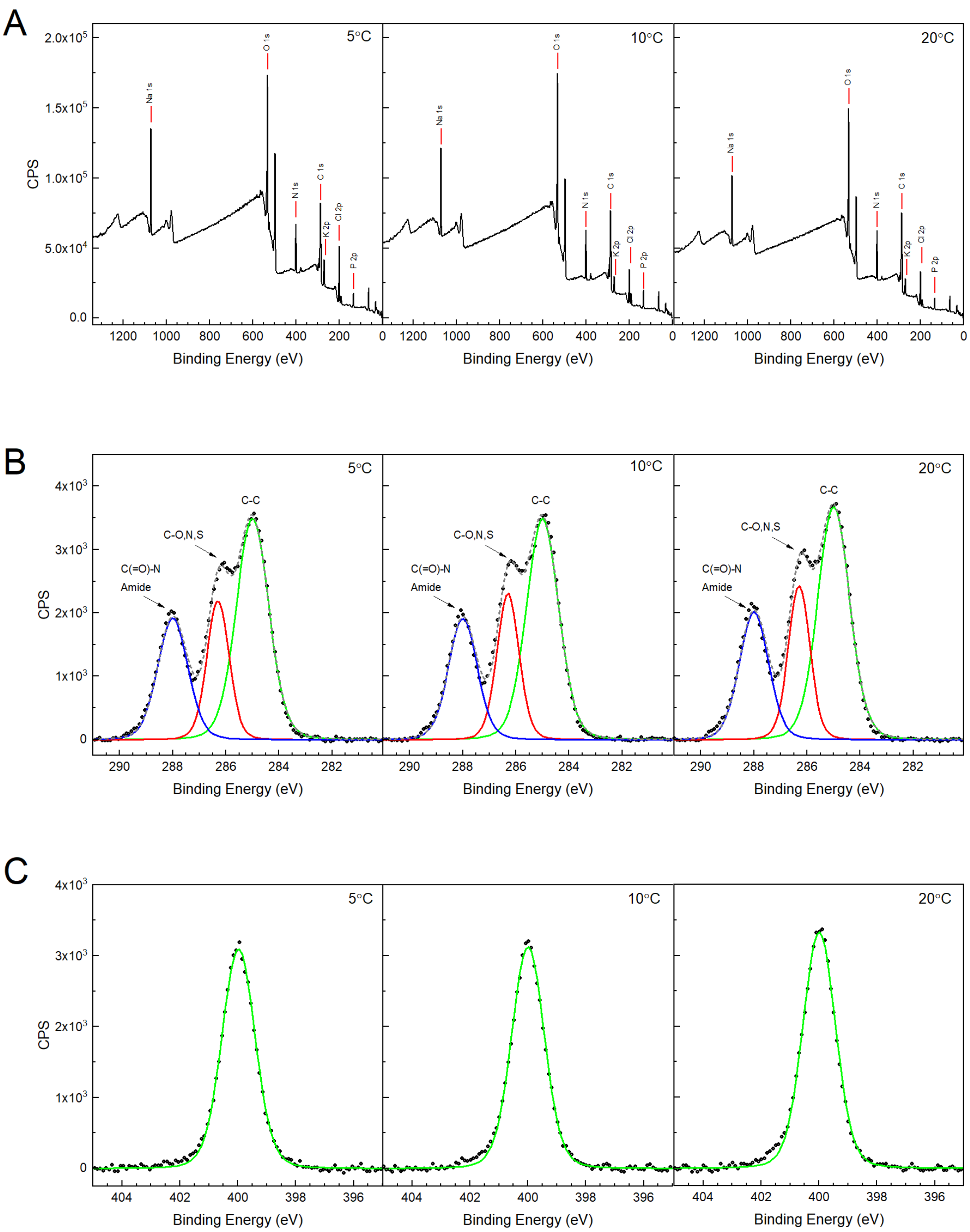

Supplementary Figure 1. XPS spectra collected from the lift-off of protein INP9R on silica at various temperatures *of A) survey scans, B) C1s high-resolution spectra, and C) N1s high-resolution spectra. The C1s high-resolution spectra have peaks at 285.0, 286.4, and 288.0 eV corresponding to C-C, C-O, N, or S, and carbon in an amide bond, respectively. The N1s high-resolution spectra have one peak at 400.0 eV corresponding to nitrogen in an amide bond.*

***Supplementary Table 1. XPS elemental compositions determined from survey scan of lift-off experiments of INP9R** at the air-water interface with varying temperature (PBS buffer subphase).*

Element	INP9R 5˚C [%]	INP9R 10˚C [%]	INP9R 20˚C [%]
**O_{1s}**	22.9 (0.4)	25.3 (0.4)	22.5 (0.3)
**C_{1s}**	40.6 (1.5)	40.8 (0.8)	41.1 (1.7)
**N_{1s}**	8.9 (0.3)	9.2 (0.5)	9.0 (0.7)
**Cl_{2p}**	7.7 (1.1)	3.7 (1.6)	7.9 (2.0)
**K_{2p}**	11.7 (0.2)	11.6 (0.2)	11.8 (0.3)
**Na_{1s}**	3.5 (0.4)	3.2 (0.2)	3.3 (0.6)
**S_{2p}**	0.3 (0.1)	0.2 (0.2)	0.2 (0.1)
**P_{2p}**	4.1 (0.1)	5.4 (0.4)	4.0 (0.3)
**Si_{2p}**	0.3 (0.1)	0.6 (0.6)	0.1 (0.1)

Note: ( ) = standard deviation (n=3); [%] = atomic percent

Supplementary Note 2: Spectral Calculations

Supplementary Methods 2

2D-IR Spectra

The spectra were calculated based on an amide-I Hamiltonian model.[5] Briefly, we construct a one- and two-exciton Hamiltonian for the amide-I mode of the backbone amide groups in the protein, with couplings that are estimated differently for nearest- and nonnearest-neighbor amide groups. The nearest-neighbor interactions, which are dominated by through-bond effects, are modeled using a parameterized map of an *ab initio* calculation with the 6-31G+(d) basis set and the B3LYP functional, thus providing the coupling as a function of the dihedral angle.[6, 7] The nonnearest-neighbor interactions, which are dominated by through-space effects, are estimated with the transition-dipole coupling model.[8] The local-mode IR frequencies ν_{local} are shifted with the same model employed by Lu et al.[9]

$$\nu_{local} = \epsilon_0 - \alpha * (r_{C=O} - r_{C=O\ equi.})$$

The values of ϵ_0 and α, as well as the number of frames over which to average the C=O length, was calibrated to LKα_{14} data and simulations using the same force field as in the current study, and found to be 1660 cm^{-1}, 500 cm^{-1}/Å and 1, respectively. The equilibrium bond distance $r_{C=O\ equi.}$ of C=O, was set to 1.229 Å for amide groups with secondary amines, which corresponds to the equilibrium value of a C-O bond in the AMBER99SB-ILDN force field, and to 1.232 Å for amide groups with tertiary amines (prolines), for which the local-mode frequency is also redshifted by 26.3 cm^{-1} to account for the redshift due to the larger carbon mass as compared to the hydrogen mass bound to the amide N atom in other amino acids.

The Hamiltonian is then diagonalized to obtain the amide-I eigenvalues and eigenvectors, from which the spectroscopic response is calculated.

SFG Spectra

To account for the azimuthal isotropy of the proteins at the interface, we average the Euler angle ϕ from 0 to 2π. For the SFG spectral calculations, the total Lorentzian width was set to 20 cm^{-1}, in accordance with the experimentally determined visible bandwidth of 15 cm^{-1} plus an inhomogeneous broadening of 5 cm^{-1}. Furthermore, the interfacial refractive indices were all set to 1.18 in accordance with ref. [11].

The orientational SFG investigation employed in this study requires a single (frame of a) hypothetical structure with the z-axis of the molecular frame aligned along a symmetry axis (the long axis of the β-helix in this case) so that the azimuthal averaging can be performed well in the SFG calculations. We did not include the hydrogen bond-induced shifts applied in the IR calculations (on the MD trajectories), because for a single frame this often leads to spectral distortions as not all hydrogen-bonded states are probed well in a single frame. For a good match between the calculated and experimental spectra, the gas-phase frequency was set to 1645 cm^{-1}, 5 cm^{-1} redshifted with respect to the values used in previous studies in which similar calculations were performed[12, 13, 14, 15]. This redshift is probably necessary to account for the stronger hydrogen bonding within the β-helix as compared to the globular proteins investigated

in the other studies. Both for θ and Ψ we assumed a Gaussian broadening of the orientation distribution with $\sigma = 10°$, to account for the fact that the protein is not expected to have a very well-defined orientation distribution. To keep the spectral calculations as simple as possible, we chose to not include a lipid C=O contribution, but to focus on optimizing the match between the model and the experimental protein signal between 1600-1670 cm^{-1}, which is expected to be unaffected by the tail of the lipid peak (the peak centered at 1730 cm^{-1}).

The reported (θ,Ψ) values reported in the main text are determined by starting Levenberg-Marquardt fits with the minima of the 2D-RSS plots taken as the initial guesses. The errors are defined as the value for which the RSS doubles in a procedure in which the angles were fixed to values away from the optimal values, and performing fits in which the other fit parameters were left free. Under the azimuthal symmetry assumption and for small twist angles (θ), the uncertainty for the twist angle Ψ is relatively large, because for such angles Ψ becomes increasingly similar to the in-plane rotation angle ϕ.

Supplementary Results 2

The Effect of Inhomogeneous Broadening on Calculated 2DIR Spectra

In Supplementary Figure 2, the effect of the addition of inhomogeneous broadening to the calculated 2D-IR spectra is shown, along the experimental spectra. One can see that while the peak frequency is already predicted well by applying the spectral calculations to the beta-helical model, the match with the spectral shape is better with inhomogeneous broadening applied to the local-mode frequencies. In order to do this, the spectral calculation was run 50 times on the first frame of the trajectory with 50 times a gasdev[10] random distributed inhomogeneous broadening of 17.5 cm^{-1} applied. The IR calculations were performed with a Lorentzian half-width-at-half-max (hwhm) of 7.5 cm^{-1} and a Gaussian pump hwhm of 2.5 cm^{-1}. The spectra that were not inhomogeneously broadened, were calculated by averaging the IR response of 250 equispaced frames of the 10 ns trajectories (thus spaced by 40 ps).

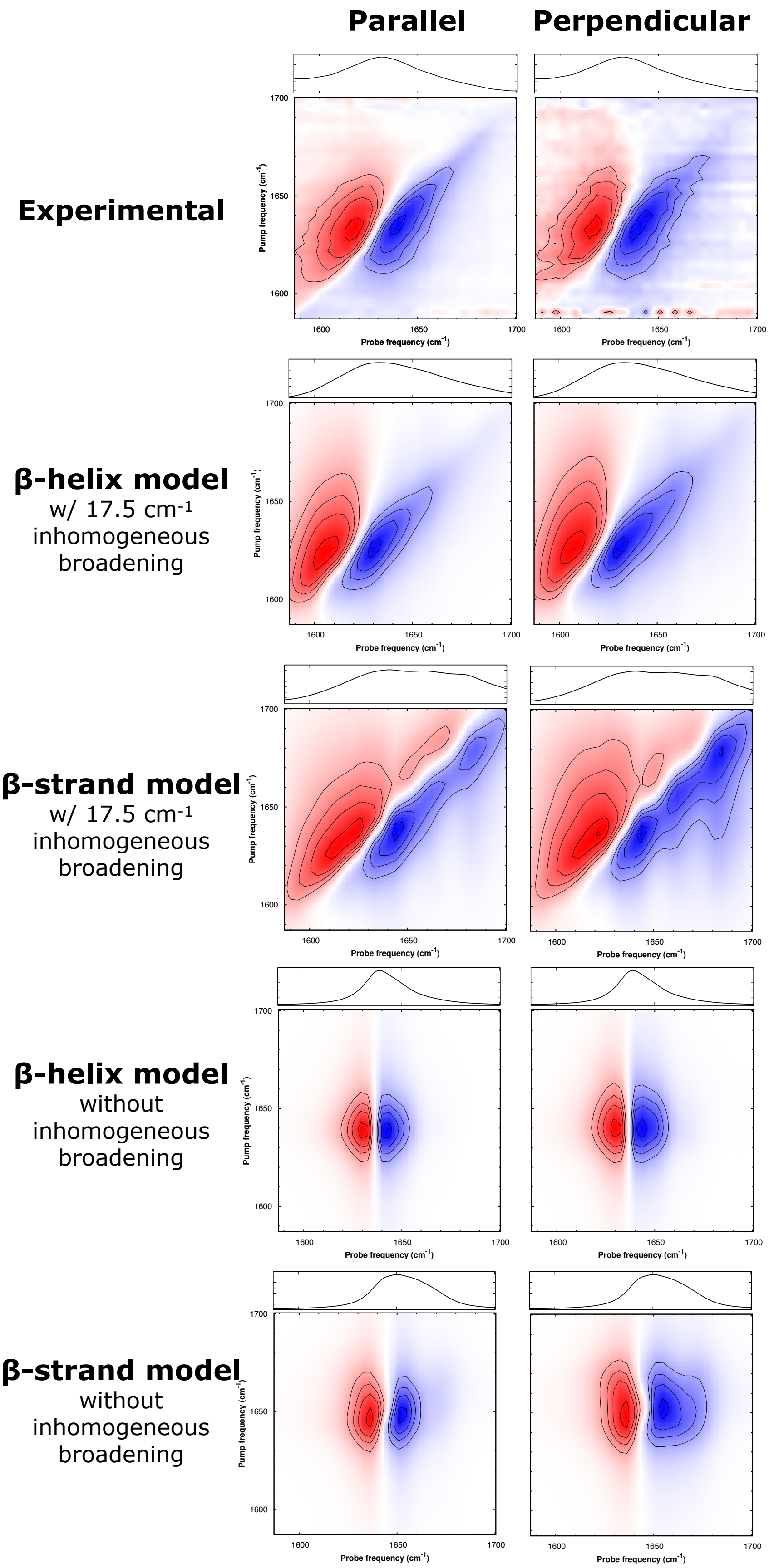

Supplementary Figure 2. Comparing experimental FT-IR and 2D-IR spectra with several methods of spectral calculations *for (in the 2D-IR case) a parallel and perpendicular pump-probe polarization. The spectra indicate that the β-helix model leads to the closest match with the experimental spectra, both with and without a random-coil contribution added to the calculations in the form of an inhomogeneous broadening of the local-mode frequencies.*

Normal-mode Analysis

In order to gain more insight into the molecular order of the observed IR and SFG response, we performed a normal-mode analysis for the low- and high-frequency normal modes that have the strongest IR response, the result of which can be found in Supplementary Figure 3.

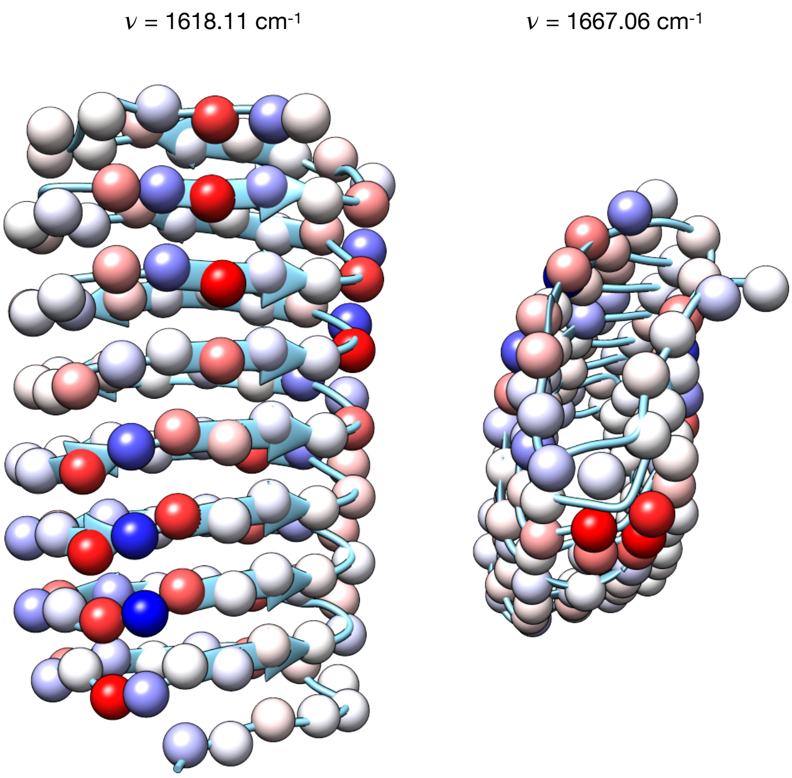

Supplementary Figure 3*. *Normal-mode analysis of the strongest low- and the strongest high-frequency modes, *revealing very close matches to the theoretically predicted B_2 and β-turn modes in ref 16 with the intensity of the colors indicating the local-mode contributions (the eigenvectors), while the blue/red color indicates the phase of the eigenvector elements. The low-frequency, B_2, mode has an opposite phase with respect to the neighboring amide-I oscillators in the strand direction, and a similar phase with respect to the neighboring amide-I groups along the β-helix' axis, while the normal-mode associated with the high-frequency mode is localized strongly on amide groups in turn regions.*

Supplementary Note 3: Sample Preparation and Characterization

Supplementary Methods 3

Biochemistry

The InaZ9R construct was prepared essentially as described previously for a 16 repeat InaZ construct.[1] Briefly, a PCR product encoding the N-terminal domain, repeats 1-4 and 63-67 and the C-terminal domain (see Supplementary Table 2 for the primer sequences) was prepared by splicing-by-overlap PCR (SOE-PCR) using a codon-optimized (Genscript) version of the 67 repeat *inaZ* gene from the *P. syringae* strain R10.79 as template as previously described[1]. The PCR product was cloned into the pET30 Ek/LIC vector according to the manufacturer's instructions (Novagen, Merck Biosciences). This generated a recombinant construct where the truncated InaZ sequence was fused with a tag-sequence containing a His-tag and an S-tag encoded by the vector and a TEV cleavage site (introduced via the cloning primers) in the N-terminal of the construct. This construct was termed InaZ9R (see the full InaZ9R sequence in main text Figure 1B and Supplementary Figure 6).

The InaZ9R plasmid was transformed into the *E. coli* Rosetta (DE3) strain and large-scale liquid cultures were grown in LB medium at 37°C until an OD600 of 0.6-0.8 and induced over night at 20°C using 1mM IPTG. The cells were lysed in lysis buffer (20 mM Tris-HCl pH 7.5; 500 mM NaCl; 20 mM Imidazole) with protease inhibitors added (cOmplete protease inhibitor cocktail, Roche). The protein was purified using immobilized metal affinity chromatography using a HisTrap FF crude column (GE Healthcare) equilibrated in lysis buffer and eluted with elution buffer (20 mM Tris-HCl pH 7.5; 500 mM NaCl; 500 mM Imidazole). Fractions containing the InaZ9R were pooled, concentrated and loaded on a MonoQ 5/50 GL column (GE Healthcare) for anion exchange chromatography. The column was equilibrated in buffer A (20 mM Tris-HCl pH 7.5; 50 mM NaCl) and eluted with a 20 column volumes of buffer B in a gradient of 0-100% (20 mM Tris-HCl pH 7.5; 1000 mM NaCl). Fractions containing the InaZ9R protein were pooled, concentrated and loaded on a Superose 6 Increase 10/300 GL column (GE Healthcare) equilibrated in SEC buffer (20 mM Tris-HCl pH 7.5; 150 mM NaCl) for size exclusion chromatography. Fractions containing InaZ9R were pooled and flash frozen in liquid nitrogen and stored at -80 ^{o}C for later use. Based on SDS-PAGE analysis (**Error! Reference source not found.**), the purified protein had a homogeneity of >90%. Constructs are available from the authors upon reasonable request.

Supplementary Table 2. Complete list of all primers used.

Name	Sequence
INP 9 repeat AB 661-714bp reverse	/5´- ACCTGCGGTCTGGTTACTGTCTGCACCTGC GGTCAGTGCGCTATCACCGCCCGCGGTCTGCGT CGAACCATAGCCAGCCACCAG -3´/
INP 9 repeat CD 3499-3548bp forward	/ 5´- GCAGGTGCAGACAGTAACCAGACCGCAGG TGATCGCAGCAAACTGCTGG -3´/
INP LIC N-terminal 4-26bp forward	/ 5´- GACGACGACAAGATG – GATTACGACATCC CCACTACT – GAGAATCTTTATTTTCAGGGC – ATGAACCTGGATAAAGCTCTGGT -3´/<br><br>(LIC overhang – linker – TEV site – 23 bp)
INP LIC C-terminal 3860-3885bp reverse	/ 5´- GAGGAGAAGCCCGGTTCA – CTA – TTCAA CTTCAATCCAATCGTCTTCTT -3´/<br><br>(LIC overhang – stop codon – 26 bp)

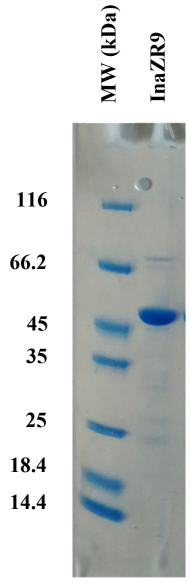

***Supplementary Figure 4**. **SDS-PAGE of purified InaZ9R.** Left lane is loaded with molecular-weight marker (with the molecular weight of the marker bands shown to the left of the gel) and right lane is loaded with the peak fraction from size-exclusion column.*

InaZ Lyophilization of the IR and SFG Samples Assessed with UV-CD

InaZ9R was lyophilized overnight in a ScanVac Coolsafe (Labogene) before all experiments, so that the SFG and IR amide-I spectra would not be affected by the H_2O bending mode (1643 cm^{-1}) that overlaps with the amide-I (1600-1700 cm^{-1}) region. Normalized UV-CD spectra before and after lyophilization indicate that the secondary structure of the protein is not affected by this procedure (see Supplementary Figure 5).

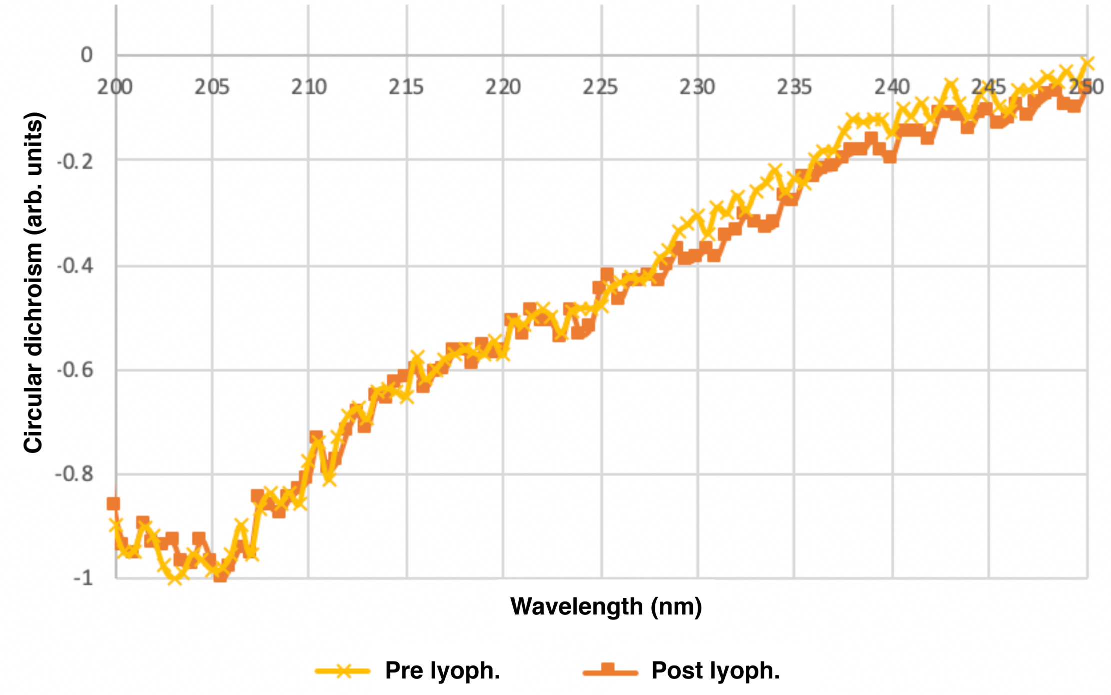

Supplementary Figure 5. Normalized UV-CD spectra of InaZ9R before and after lyophilization.

The UV-CD spectra exhibit a random coil contribution around 205 nm, as well as a broad peak around 217 nm that probably indicates β-helical contents.[2] These UV-circular dichroism (UV-CD) spectra were recorded with a Chirascan-plus CD spectropolarimeter (Applied Photophysics, Leatherhead, UK), using a 1 mm pathlength quartz cuvette. The inaZ9R was dissolved in PBS buffer at a concentration of 0.05 mg/ml. The spectra were obtained at room temperature in the spectral area of 200 nm to 250 nm set to 0.5s time-per-point, step size of 0.2 nm, and 2 nm bandwidth.

FT-IR and 2D-IR Sample Preparation

The lyophilized InaZ9R in powder form was resolvated at 0.8 mg/mL in phosphate buffered saline (PBS; 0.01 M phosphate buffer, 0.0027 M KCl, and 0.137 M NaCl, pD 7.4, Sigma–Aldrich), D_2O (99.9%D, Eurisotop), and 7 µL of the solution was placed in between two 1 mm CaF_2 windows and sealed off with a Krytox vacuum greased (Duniway) 50 µm spacer in a custom-made IR cell.

SFG Sample Preparation

The samples for SFG measurements were prepared from lyophilized inaZ9R powder in D_2O-PBS buffer to avoid interference from H_2O bending modes. The samples were prepared in a stainless-steel trough with a quartz window at the bottom containing approximately 3 mL of solution. Throughout all SFG experiments, the water level was held constant by a syringe pump (New Era Pump Systems Inc.) with the cannula submerged to the bottom of the trough. A solution of D_2O-PBS (pD = 7.4) with inaZ9R was added to the trough such that the final protein concentration was 10 μM. Throughout the experiment, a water chiller (Neslab RTE-101) was used with a water-cooled breadboard to adjust the temperature of the protein solution to 20°C, 10°C, and 5°C, as monitored with a submerged digital thermometer (Omega).

Supplementary Results 3

Mass Spectral Analysis of Recombinant InaZ

The recombinant InaZ9R produced was assayed by mass spectrometry to confirm protein sequence and (denatured) holo-mass of the produced protein in comparison to the nucleotide sequence of the construct. This can reveal unexpected post-translational and chemical modifications as well as amino-acid substitutions. In addition, we assayed the protein by native-spray mass spectrometry to determine possible complex formation and different folding states of the protein.

To this end, the purified protein was subjected to digest by trypsin with chymotryptic activity (owing to the limited number of fully tryptic sites in the protein) and analyzed by data dependent tandem mass spectrometry on a timsTOFpro mass spectrometer (Bruker Daltonik, Germany). Data were analyzed by fragment ion searches performed by an in-house MASCOT server (matrix science, UK) against the sequence of InaZ alone and in the background of the *Escherichia coli* proteome database, the latter to assay the preparation for background proteins stemming from the recombinant production of the protein (see Supplementary Figure 6). Searches were conducted with cysteine carbidomethylation and methionine oxidation set as fixed and variable modifications respectively, against the InaZ sequence. This showed a maximum of 56% sequence coverage of the proposed sequence, which, owing to the limited number of fully tryptic cleavage sites, is a good coverage for this protein. In addition, the data was searched against the same InaZ sequence in the background of the *E. coli* proteome, to

assay for purity of the preparation, InaZ was the major protein found in the sample (47% coverage), with a number of background proteins from *E. coli* also identified (minor components). To assay for possible unexpected modifications of InaZ, we also analyzed the data using PEAKS studio X+ (BSI bioinformatics) using its *de novo* search algorithm and found no indication of unexpected post-translational modifications to the protein (data not shown).

Protein sequence coverage: 56%

Matched peptides shown in ***bold red***.

1 MHHHHHHSSG LVPRGSGMLE TAAALFERQH MDSPDLGTDD DDKEN**LYFQG**
51 **MNLDK**ALVLR TCAN**NMADHC** **GLIWPASGTV** **ESK**YWQSTRR HENGLVGLLW
101 **GAGTSAFLSV** **HADARWIVCE** **VAVADIITLE** **EPGMVK**FPR**A** **EVVHVGDRIS**
151 **ASHFISARQA** **DPASTPTPTP** **TPMA**TPTPTP AAANVALPVA EQASHEVFDV
201 ALVSAAAPPV NTLPVTTPQN LQTAT**YGSTL** **SGDNHSRLIA** **GYGSNETAGN**
251 **HSD**LIAGYGS TGTAGSDSSL VAGYGSTQTA GGDSALTAGA DSNQTAGDR**S**
301 **KLLAGNNSYL** **TAGDRSKLTG** **GHDCTLMAGD** **QSR**LTAGK**NS** **VLTAGARSKL**
351 **IGSEGSTLSA** **GEDSTLIFR**L WDGKR**YRQLV** **ARTGENGVEA** **DIPYYVNEDD**
401 **DIVDKPDEED** **DWIEVE**

Supplementary Figure 6. Sequence coverage of InaZ by tryptic and chymotryptic digest. *Purified InaZ subjected to digestion by trypsin and chymotrypsin was analyzed by mass spectrometry. Peptide sequences identified ($p<0.05$) are shown in bold red in the protein sequence.*

In addition to bottom-up analysis of tryptic digests, we also assayed the intact protein by denatured and native spray mass spectrometry on a SynaptG2 mass spectrometer (Waters, UK). In short, InaZ was diluted in 50% acetonitrile, 49.9% water, 0.1% formic acid to an approximate concentration of 1 µM, and sprayed into the mass spectrometer by loading the solution into a glass-tip emitter (New Objective, USA). The mass spectrometer was externally calibrated for a range of 500-5000 m/z by sodium cesium iodide clusters and source settings were as follows: capillary voltage 1200 V, cone voltage 45 V, source gas 0.3 bar. The raw spectrum is shown in Supplementary Figure 7 and, although noisy, we are able to extract numerous components with a mass around 45 kDa (expected mass 43.834 kDa), which is highly similar to the mass of ~45 kDa observed on SDS page. To ascertain whether InaZ might form different folding structures with differing charge states or form complexes with itself in solution, we also attempted to measure InaZ under native conditions, using the same source settings. In this case, InaZ was diluted to approximately 1 µM in 1 mM ammonium acetate at pH 5.0. This, however, resulted in a very undefined mass spectrum (data not shown) that could not be interpreted. This could be due to the heterogeneity already shown in the poorly defined denatured spectrum being exacerbated by multiple folding or aggregation states of the protein under native conditions.

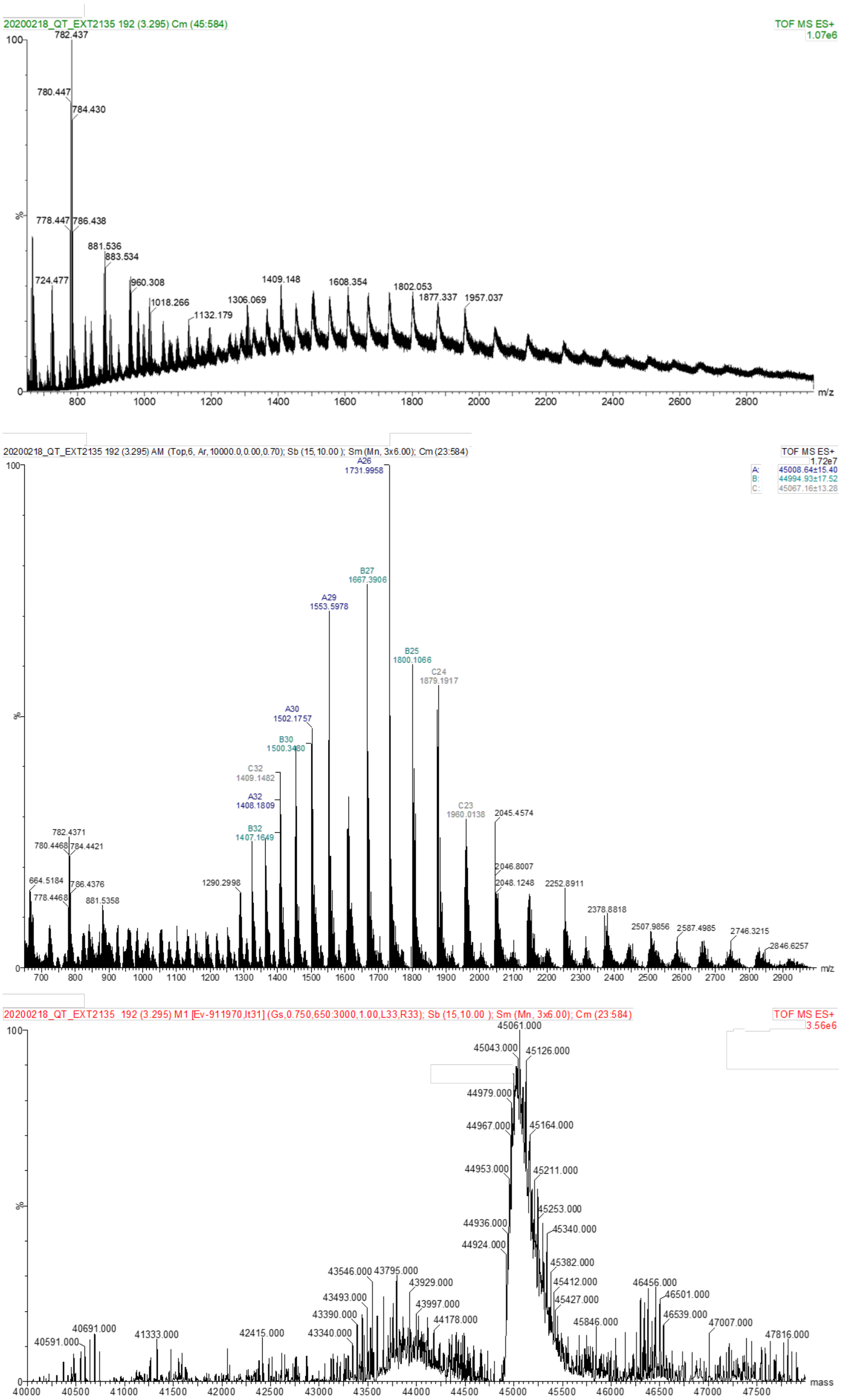

Supplementary Figure 7. Mass spectra of intact inaZ. *The top panel shows the mass spectrum acquired from intact InaZ diluted in 50% acetonitrile, 49.9% water and 0.1% formic acid. The middle panel shows the processed spectrum (smoothed, background subtracted and centered masses) in which various components are annotated manually by the component extraction function of masslynx 3.4 (Waters, UK). Owing to the relatively noisy spectra, various components can be extracted with varying degrees of mass accuracy around a mass of 45 kDa. The bottom panel shows the automated deconvolution of the top spectrum using the MaxEnt-I algorithm of masslynx, which agrees with the manually annotated components showing a broad peak around 45 kDa with only a poorly defined component around the expected mass of 43 kDa.*

Supplementary Note 4: 1D-IR (FT-IR) and 2D-IR Spectroscopy

Supplementary Methods 4

1D-IR (FT-IR) Spectroscopy

The FTIR spectra were recorded with 32 scans on a Bruker Vertex v70 spectrometer. The sample compartment was purged with N_2 gas to avoid absorption lines due to water lines.

2D-IR Spectroscopy

For the 2D-IR experiment, 794 nm pulses were generated with a Mantis (Coherent) oscillator at an 80 MHz rate, of which 1 kHz were amplified with a Legend (Coherent) optical parametric amplifier to a 3 mJ beam. This light is converted to ~15 µJ of mid-IR (~6100 nm) light, with a full-width-at-half-max (fwhm) of ~150 cm^{-1}, after which it is split into a probe, reference and pump beam at a 5/5/95% ratio. The pump beam is spectrally narrowed to a fwhm of 10 cm^{-1} by a Fabry-Pérot interferometer and overlapped in space with a 1.5 ps delay in the sample with the probe beam, while the reference beam passes through the sample cell a few mm beside the focus of the probe and pump. The difference absorbance spectrum is then dispersed by an OrielMS260i spectrograph onto a 32 pixel MCT array with a resolution of 3.9 cm^{-1} (see supplementary reference 3 for more details).

Supplementary Results 4

Temperature-Dependent IR Spectra

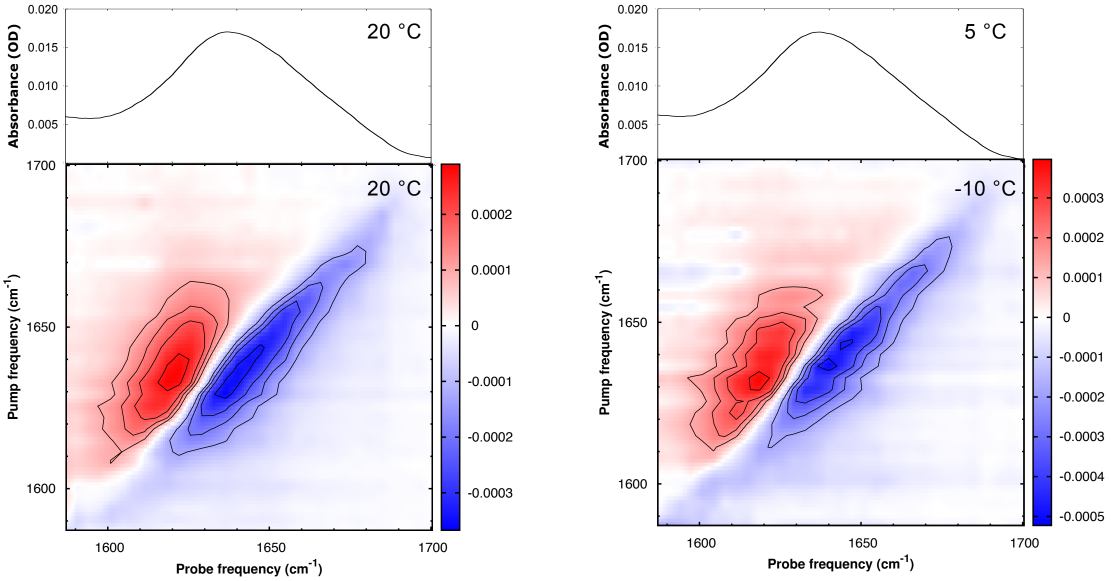

Supplementary Figure 8. Temperature-dependent 1D-IR (FT-IR, top) and 2D-IR (bottom, with the color bars indicating the differential absorption in ΔOD) spectra *at different temperatures for an InaZ9R concentration of 4.3 mg/mL in PBS prepared with D_2O, and with a parallel pump polarization with respect to the probe polarization.*

In Supplementary Figure 8 one can see that the secondary structure does not change for a similar temperature change as employed in the SFG experiments (from +20 °C to 5 °C, on top). Even when going down to -10 °C (bottom) the amide-I spectrum does not change significantly. The minor spectral changes are probably a result of scattering induced by vapor formation due to the low temperature, on the outer side of the CaF_2 windows of the IR cell. The sample does not yet freeze at this temperature, probably due to the very small sample volume (7 µL) and the presence of the salt in the PBS buffer.

Supplementary Note 5: SFG Spectroscopy

Supplementary Methods 5

SFG Spectroscopy

The SFG setup is based on a 7 W, 35 femtosecond laser system (Astrella, Coherent) with pulses centered at 800 nm and a repetition rate of 1 kHz. One part of the output was used to pump an optical parametric amplifier (OPA) with a non-collinear difference frequency generation (NDFG) extension (TOPAS Prime, Light Conversion) to generate broadband (FWHM ~ 300 cm^{-1}) IR pulses tunable over the range 3.3-6.1 µm. A narrowband (FWHM ~ 15 cm^{-1}) visible beam was generated by guiding 1 mJ of the fundamental through a Fabry-Perot etalon. The visible and IR beams were spatially and temporally overlapped on the surface. The SFG signal was focused into a spectrograph (Shamrock 303i, Andor) and detected by an EMCCD camera (Newton 971, Andor). In the present study, SFG measurements were recorded in the amide I region (1600-1700 cm^{-1}), O-D stretching region (2200-2800 cm^{-1}), and C-H stretching region (2800-3100 cm^{-1}). Spectra in the Amide I region were collected in ssp (s-SFG, s-visible, p-IR) and ppp polarization combinations. Spectra in the O-D region were recorded in ssp polarization combination and spectra in the CH region were recorded in ssp and sps polarization combinations. The sample stage and IR beam path were flushed with nitrogen to avoid artifacts due to IR light adsorption by water vapor. All spectra were background subtracted and normalized using a reference spectrum obtained from gold.

SFG Fitting Methods

The SFG data in the OD region were fit using the following equation:

$$\chi_{eff}^{(2)}(\omega) = \chi_{NR}^{(2)} + \sum_q \frac{A_q}{\omega - \omega_q + i\Gamma_q} \qquad (1)$$

where Γ_q, A_q, and ω_q are the full width half max (FWHM), amplitude, and resonant frequency of the qth vibrational mode, respectively, and $\chi_{NR}^{(2)}$ and $\chi_{eff}^{(2)}$ are the nonresonant background and effective seconded nonlinear susceptibility tensor, respectively. To determine the error in the measurements, the amplitude was allowed to change, and the other fitting components are held the same (this is done to directly compare the amplitude change). We determine the error for the amplitudes by allowing the amplitudes to change until the fit became unreasonable. This error was determined to be 10 percent.

Supplementary Results 5

SFG Spectra in the C-H Region

Supplementary Figure 9 depicts the C–H stretch region SFG spectra of inaZ9R at the air–water interface at temperatures of 20 °C (left) and 5 °C (right). The observed C-H spectra are indicative of protein side chains and resonances are observed near 2850, 2870, 2910, 2935, and 2950 cm^{-1}. These are assigned to CH_2 symmetric, CH_3 symmetric, CH_2 asymmetric, CH_3 Fermi, and CH_3 asymmetric resonances, respectively. The observed increase in overall intensity upon decreasing the temperature from 20 °C to 5 °C, can be attributed to inaZ9R ordering or changing orientation at the interface as temperature decreases.

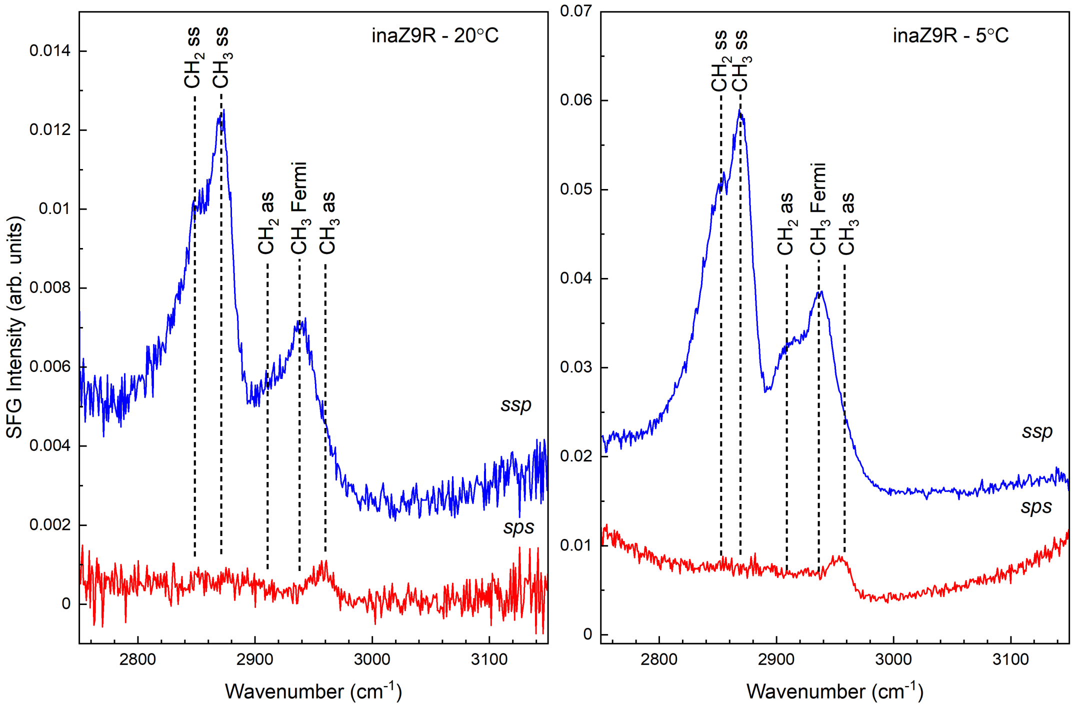

Supplementary Figure 9. SFG spectra of inaZ9R at the air–D_2O interface in the C-H strecth region.

Peak-Fitting Parameters of the OD-Stretch Spectra

The parameters obtained from fitting the SFG data from Figure 2 of the main text are shown below in Supplementary Table 3 (for the spectra with inaZ9R) and Supplementary Table 4 (for the spectra without inaZ9R).

Supplementary Table 3. SFG spectral parameters for the OD region: air–D_2O PBS interface with inaZ9R.

Sample/polarization	A_{NR}	φ_{NR}	ω_n (cm^{-1})	Γ_n (cm^{-1})	A_n	assignment
inaZ9R 20°C *SSP*	0.006	5.53	2363	84	4.397 (± 0.440)	O-D stretch[28, 29]
			2408	123	6.829 (± 0.683)	O-D stretch when a bio-molecule such as a protein or lipid is present[29]
			2485	130	6.922 (± 0.738)	O-D stretch[28]
			2643	32	0.028 (± 0.003)	From free O-D and protein interaction[29]
inaZ9R 10°C *SSP*	0.017	5.53	2363	84	5.537 (± 0.554)	O-D stretch[28, 29]
			2408	123	7.938 (± 0.794)	O-D stretch when a bio-molecule such as a protein or lipid is present[29]
			2485	130	9.620 (± 0.962)	O-D stretch[28]

			2643	32	0.081 (± 0.008)	From free O-D and protein interaction[29]
inaZ9R 5℃ *SSP*	0.005	5.53	2363	84	7.553 (± 0.755)	O-D stretch[28, 29]
			2408	123	10.550 (± 1.055)	O-D stretch when a bio-molecule such as a protein or lipid is present[29]
			2485	130	11.274 (± 1.127)	O-D stretch[28]
			2643	32	0.139 (± 0.014)	From free O-D and protein interaction[29]

Supplementary Table 4. SFG spectral parameters for OD region: neat air–D_2O PBS interface.

Sample/polarization	A_{NR}	φ_{NR}	ω_n (cm^{-1})	Γ_n (cm^{-1})	A_n	assignment
D_2O-PBS 20℃ *SSP*	0.041	6.32	2375	160	7.583 (± 0.758)	O-D stretch[28]
			2460	105	1.902 (± 0.190)	O-D stretch[28]
			2682	133	3.467 (± 0.347)	Free O-D[28] and salt interaction
D_2O-PBS 10℃ *SSP*	0.062	6.32	2370	170	7.804 (± 0.780)	O-D stretch[28]
			2480	140	3.047 (± 0.305)	O-D stretch[28]
			2670	190	5.123 (± 0.512)	Free O-D[28] and salt interaction
D_2O-PBS 5℃ *SSP*	0.031	6.32	2370	170	7.508 (± 0.751)	O-D stretch[28]
			2480	140	3.586 (± 0.359)	O-D stretch[28]
			2670	190	5.439 (± 0.544)	Free O-D[28] and salt interaction

Peak-Fitting Parameters of the Amide-I Spectra

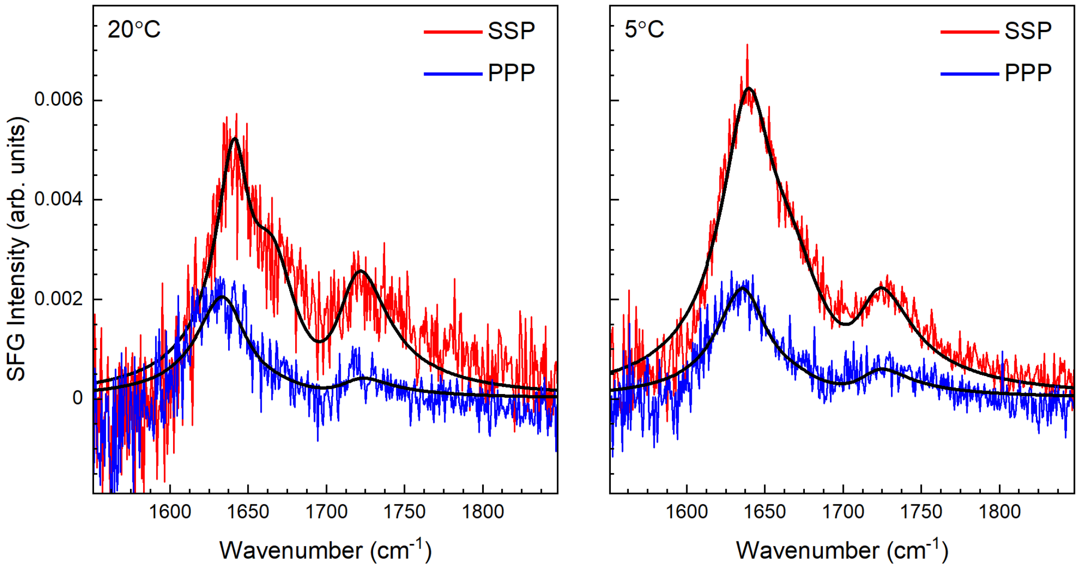

Supplementary Figure 10. Fits of InaZ at the air–water interface at two different temperatures.

Supplementary Table 5*. SFG fit of amide I spectra*

Sample	ω_n (cm-1)	Υ_n (cm-1)	A_n
20° C InaZ SSP	1643	24.97	0.541
	1665	41.61	0.876
	1718	39.00	0.738
20° C InaZ PPP	1635	38.90	0.791
	1665	57.89	0.191
	1718	39.52	0.252
5° C InaZ SSP	1641	38.64	1.011
	1665	57.89	1.040
	1720	40.72	0.550
5° C InaZ PPP	1637	38.90	0.781
	1665	57.89	0.291
	1720	39.31	0.305

Note: The peak positions near 1636 and 1668 cm^{-1} can be assigned to B_2 mode and β-turns/unordered structures of a β-structure protein[16], and the 1720 cm^{-1} peak can be assigned the lipid ester mode[30]. These assignments are also found back in the normal-mode analysis derived for these peaks (see Supplementary Figure 3).

Supplementary Note 6: Molecular-Dynamics Simulations

Supplementary Methods 6

Two hypothetical INP structures were used in this MD study, a stacked antiparallel β-sheet model obtained from Andrey Kajava, and a β-helical model obtained from Peter Davies. The stacked antiparallel β-sheet model was first simulated in a 4.7 x 5.1 x 5.7 nm^3 water box containing 4337 water molecules and 3 sodium counterions. The β-helix model was simulated in a 6.4 x 5.2 x 4.2 nm^3 water box containing 4066 water molecules and 8 sodium counterions. All simulations were performed using GROMACS 2019.[31] using the GROMOS-53A6 forcefield[32] in combination with the SPC/Ewater model.[33] First each system underwent a steepest descent energy minimization to remove any unfavorable contacts, with a tolerance of 1000 kJ mol^{-1} nm^{-1}. Following this, the systems were equilibrated to 300 K (26.85 °C) using a stochastic velocity-rescaling thermostat[34] ($\tau = 0.1$ ps) and to 1 bar using a Parrinello-Rahman barostat[35] ($\tau = 1.0$ ps) over 100 ps. In all simulations, periodic boundaries were applied in all directions. All simulations were run using a timestep of 2 fs, and hydrogen bonds were constrained in all simulations by the LINCS algorithm.[36] Electrostatic interactions were calculated with the particle mesh Ewald (PME) summation method.[37] A van der Waals cutoff value of 1.0 nm was used.

Following equilibration, production simulations were carried out in solution in the NVT ensemble at two different temperatures 300K (26.85 °C), and 278K (5 °C) using the same parameters used for equilibration.

Supplementary Results 6

The production run was carried out for 10 ns to assess the stability of the proposed structures in solution at both temperatures. The root-mean square deviation of the alpha-carbon atoms is depicted in Supplementary Figure 11.

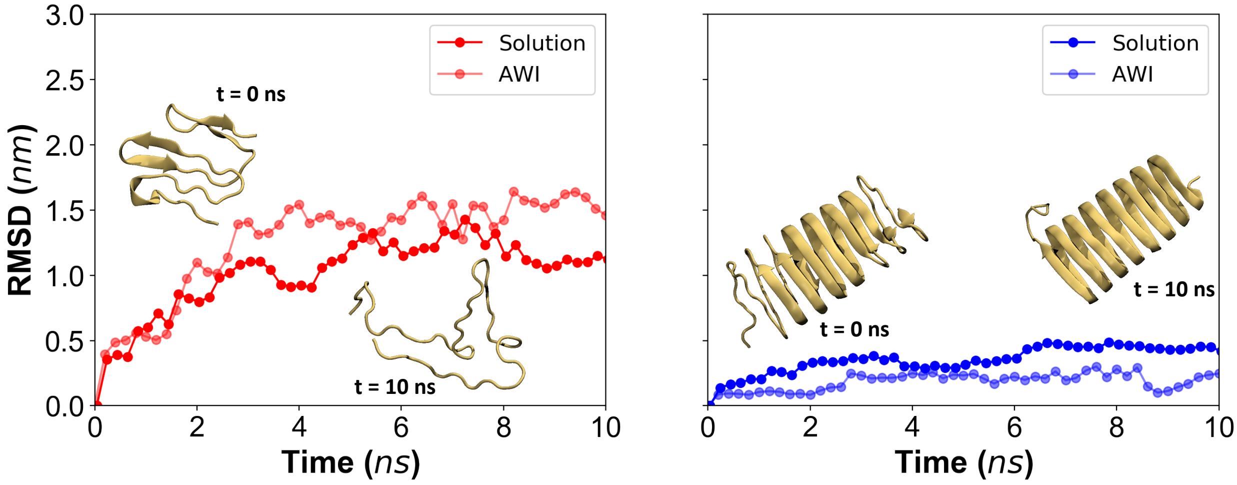

Supplementary Figure 11. Stability of (left) stacked antiparallel β-sheet and (right) β-helix model *monitored by taking the RMSD of the α-carbon in reference to frame 0.*

Supplementary References

1. Ling M*, et al.* Effects of ice nucleation protein repeat number and oligomerization level on ice nucleation activity. *Journal of Geophysical Research: Atmospheres* **123**, 1802-1810 (2018).

2. Sieber V, Jurnak F, Moe GR. Circular dichroism of the parallel β helical proteins pectate lyase C and E. *Proteins: Structure, Function, and Bioinformatics* **23**, 32-37 (1995).

3. Huerta-Viga A, Shaw DJ, Woutersen S. pH dependence of the conformation of small peptides investigated with two-dimensional vibrational spectroscopy. *The Journal of Physical Chemistry B* **114**, 15212-15220 (2010).

4. Golbek TW, Schmüser L, Rasmussen MH, Poulsen TB, Weidner T. Lasalocid Acid Antibiotic at a Membrane Surface Probed by Sum Frequency Generation Spectroscopy. *Langmuir* **36**, 3184-3192 (2020).

5. Roeters SJ*, et al.* Determining In Situ Protein Conformation and Orientation from the Amide-I Sum-Frequency Generation Spectrum: Theory and Experiment. *J Phys Chem A* **117**, 6311–6322 (2013).

6. Hamm P, Zanni M. *Concepts and Methods of 2D Infrared Spectroscopy*. Cambridge University Press, Cambridge, U.K. (2011).

7. Gorbunov RD, Kosov DS, Stock G. Ab initio-based exciton model of amide I vibrations in peptides: Definition, conformational dependence, and transferability. *J Chem Phys* **122**, 224904 (2005).

8. Krimm S, Bandekar J. Vibrational spectroscopy and conformation of peptides, polypeptides, and proteins. In: *Advances in protein chemistry* (ed^(eds). Elsevier (1986).

9. Lu H*, et al.* Peptide-Controlled Assembly of Macroscopic Calcium Oxalate Nanosheets. *The Journal of Physical Chemistry Letters* **10**, 2170-2174 (2019).

10. Press WH, Teukolsky SA, Vetterling WT, Flannery BP. Numerical recipes in C. (ed^(eds). Cambridge university press Cambridge (1988).

11. Zhuang X, Miranda P, Kim D, Shen Y. Mapping molecular orientation and conformation at interfaces by surface nonlinear optics. *Physical Review B* **59**, 12632 (1999).

12. Bernhard C, Roeters SJ, Franz J, Weidner T, Bonn M, Gonella G. Repelling and ordering: the influence of poly(ethylene glycol) on protein adsorption. *Phys Chem Chem Phys* **19**, 28182–28188 (2017).

13. Roeters SJ, Tronic EH, Baio JE, Castner DG, Weidner T. Structure of von Willebrand factor A1 on polystyrene determined from experimental and calculated sum frequency generation spectra. *Biointerphases* **13**, 06E411 (2018).

14. Verreault D, Alamdari S, Roeters SJ, Pandey R, Pfaendtner J, Weidner T. Ice-binding site of surface-bound type III antifreeze protein partially decoupled from water. *Phys Chem Chem Phys* **20**, 26926–26933 (2018).

15. Schmüser L, Roeters S, Lutz H, Woutersen S, Bonn M, Weidner T. Determination of Absolute Orientation of Protein α-Helices at Interfaces Using Phase-Resolved Sum Frequency Generation Spectroscopy. *The Journal of Physical Chemistry Letters* **8**, 3101-3105 (2017).

16. Nguyen KT, King JT, Chen Z. Orientation Determination of Interfacial β-Sheet Structures in Situ. *The Journal of Physical Chemistry B* **114**, 8291-8300 (2010).

17. Baio JE, Weidner T, Castner DG. *Characterizing the Structure of Surface-Immobilized Proteins: A Surface Analysis Approach* (2012).

18. Apte JS, Collier G, Latour RA, Gamble LJ, Castner DG. XPS and ToF-SIMS Investigation of α-Helical and β-Strand Peptide Adsorption onto SAMs. *Langmuir* **26**, 3423-3432 (2010).

19. Vickerman JC, Gilmore IS. *Surface Analysis: The Principal Techniques*. Wiley (2011).

20. Franz J*, et al.* Full membrane spanning self-assembled monolayers as model systems for UHV-based studies of cell-penetrating peptides. *Biointerphases* **10**, 019009 (2015).

21. Zorn G, Dave SR, Weidner T, Gao X, Castner DG. Direct characterization of polymer encapsulated CdSe/CdS/ZnS quantum dots. *Surface Science* **648**, 339-344 (2016).

22. Rafati A, Shard AG, Castner DG. Multitechnique characterization of oligo(ethylene glycol) functionalized gold nanoparticles. *Biointerphases* **11**, 04B304 (2016).

23. Baio JE, Weidner T, Ramey D, Pruzinsky L, Castner DG. Probing the Orientation of Electrostatically Immobilized Cytochrome C by Time of Flight Secondary Ion Mass Spectrometry and Sum Frequency Generation Spectroscopy. *Biointerphases* **8**, 18 (2013).

24. Baio JE, Weidner T, Baugh L, Gamble LJ, Stayton PS, Castner DG. Probing the Orientation of Electrostatically Immobilized Protein G B1 by Time-of-Flight Secondary Ion Spectrometry, Sum Frequency Generation, and Near-Edge X-ray Adsorption Fine Structure Spectroscopy. *Langmuir* **28**, 2107-2112 (2012).

25. Baio J, Weidner T, Brison J, Graham D, Gamble LJ, Castner DG. Amine terminated SAMs: Investigating why oxygen is present in these films. *Journal of electron spectroscopy and related phenomena* **172**, 2-8 (2009).

26. Techane SD, Gamble LJ, Castner DG. Multitechnique Characterization of Self-Assembled Carboxylic Acid-Terminated Alkanethiol Monolayers on Nanoparticle and Flat Gold Surfaces. *The Journal of Physical Chemistry C* **115**, 9432-9441 (2011).

27. Bednar RM*, et al.* Immobilization of Proteins with Controlled Load and Orientation. *ACS Applied Materials & Interfaces* **11**, 36391-36398 (2019).

28. Sovago M, Campen RK, Wurpel GWH, Müller M, Bakker HJ, Bonn M. Vibrational Response of Hydrogen-Bonded Interfacial Water is Dominated by Intramolecular Coupling. *Physical Review Letters* **100**, 173901 (2008).

29. Bonn M, Bakker HJ, Tong Y, Backus EHG. No Ice-Like Water at Aqueous Biological Interfaces. *Biointerphases* **7**, 20 (2012).

30. Dreier LB, Bonn M, Backus EHG. Hydration and Orientation of Carbonyl Groups in Oppositely Charged Lipid Monolayers on Water. *The Journal of Physical Chemistry B* **123**, 1085-1089 (2019).

31. Abraham MJ*, et al.* GROMACS: High performance molecular simulations through multi-level parallelism from laptops to supercomputers. *SoftwareX* **1**, 19-25 (2015).

32. Oostenbrink C, Villa A, Mark AE, Van Gunsteren WF. A biomolecular force field based on the free enthalpy of hydration and solvation: the GROMOS force-field parameter sets 53A5 and 53A6. *Journal of computational chemistry* **25**, 1656-1676 (2004).

33. Berendsen H, Grigera J, Straatsma T. The missing term in effective pair potentials. *J Phys Chem* **91**, 6269-6271 (1987).

34. Bussi G, Donadio D, Parrinello M. Canonical sampling through velocity rescaling. *The Journal of chemical physics* **126**, 014101 (2007).

35. Parrinello M, Rahman A. Polymorphic transitions in single crystals: A new molecular dynamics method. *Journal of Applied physics* **52**, 7182-7190 (1981).

36. Hess B, Bekker H, Berendsen HJ, Fraaije JG. LINCS: a linear constraint solver for molecular simulations. *Journal of computational chemistry* **18**, 1463-1472 (1997).

37. Essmann U, Perera L, Berkowitz ML, Darden T, Lee H, Pedersen LG. A smooth particle mesh Ewald method. *The Journal of chemical physics* **103**, 8577-8593 (1995).
